# Ce(iv)-centered charge-neutral perovskite layers topochemically derived from anionic [CeTa_2_O_7_]^−^ layers†

**DOI:** 10.1039/d1sc03053a

**Published:** 2021-10-15

**Authors:** Takuya Hasegawa, Naoki Yamasaki, Yusuke Asakura, Tadaharu Ueda, Shu Yin

**Affiliations:** Institute of Multidisciplinary Research for Advanced Material (IMRAM), Tohoku University 2-1-1 Katahira, Aoba-ku Sendai 980-8577 Japan hase@tohoku.ac.jp +81-22-217-5598 +81-22-217-5598; Department of Marine Resource Science, Faculty of Agriculture and Marine Science, Kochi University Nankoku 783-8502 Japan; Center for Advanced Marine Core Research, Kochi University Nankoku 783-8520 Japan

## Abstract

Layered perovskites have been extensively investigated in many research fields, such as electronics, catalysis, optics, energy, and magnetics, because of the fascinating chemical properties that are generated by the specific structural features of perovskite frameworks. Furthermore, the interlayers of these structures can be chemically modified through ion exchange to form nanosheets. To further expand the modification of layered perovskites, we have demonstrated an advance in the new structural concept of layered perovskite “charge-neutral perovskite layers” by manipulating the perovskite layer itself. A charge-neutral perovskite layer in [Ce^IV^Ta_2_O_7_] was synthesized through a soft chemical oxidative reaction based on anionic [Ce^III^Ta_2_O_7_]^−^ layers. The Ce oxidation state for the charge-neutral [Ce^IV^Ta_2_O_7_] layers was found to be tetravalent by X-ray absorption fine structure (XAFS) analysis. The atomic arrangements were determined through scattering transmission electron microscopy and extended XAFS (EXAFS) analysis. The framework structure was simulated through density functional theory (DFT) calculations, the results of which were in good agreement with those of the EXAFS spectra quantitative analysis. The anionic [Ce^III^Ta_2_O_7_]^−^ layers exhibited optical absorption in the near infrared (NIR) region at approximately 1000 nm, whereas the level of NIR absorption decreased in the [Ce^IV^Ta_2_O_7_] charge-neutral layer due to the disappearance of the Ce 4f electrons. In addition, the chemical reactivity of the charge-neutral [Ce^IV^Ta_2_O_7_] layers was investigated by chemical reduction with ascorbic acid, resulting in the reduction of the [Ce^IV^Ta_2_O_7_] layers to form anionic [Ce^III^Ta_2_O_7_]^−^ layers. Furthermore, the anionic [Ce^III^Ta_2_O_7_]^−^ layers exhibited redox activity which the Ce in the perovskite unit can be electrochemically oxidized and reduced. The synthesis of the “charge-neutral” perovskite layer indicated that diverse features were generated by systematically tuning the electronic structure through the redox control of Ce; such diverse features have not been found in conventional layered perovskites. This study could demonstrate the potential for developing innovative, unique functional materials with perovskite structures.

## Abstract 2

This study proposed a new layer modification technique, “layer charge control”, for layered perovskites, and the structures of the obtained charge neutral [CeTa_2_O_7_] perovskite sheet were characterized theoretically and experimentally.

## Graphical abstract

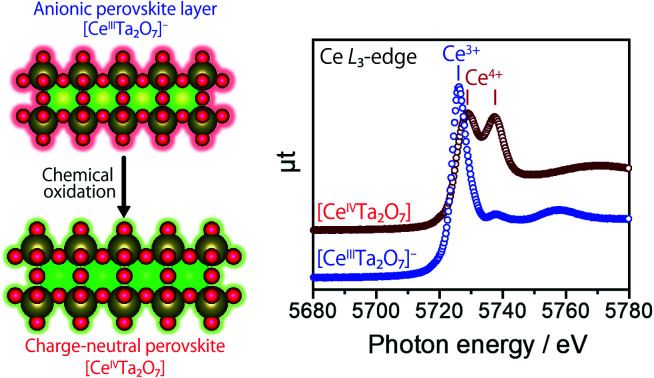

## Introduction

Extensive attention has been given to perovskite compounds in electronic, catalytic, optical and magnetic research fields due to their diverse chemical properties. Many functional inorganic compounds based on perovskite structures have been reported.^1–3^ Among various types of derived perovskite structures, layered perovskites, particularly with a two-dimensional perovskite framework containing interlayer alkali-metal cations, can be modified based on the precise design of the atomic arrangements with post-treatment; such post-treatment methods include ion exchange with other cations and exfoliation into a monolayer to add further functionality.^4–6^ A wide variety of interlayer modifications for layered perovskites have been carried out, and mixed-anion layered perovskites have been shown to induce fascinating functions and realize excellent levels of performance;^7–10^ nevertheless, limited studies on perovskite layer post-modifications, which could change the corresponding functionalities, have been conducted through anion substitutions that are fundamentally based on conventional processes similar to inorganic compound substitutions in other structures. It is quite difficult to substitute the cation parts containing multivalent ions in the perovskite framework through post-treatment. To expand the potential functionalities of layered perovskites, novel approaches have been needed for perovskite modifications beyond substitution-type techniques.

We focused on a strategy for valence tuning of perovskite layers utilizing the oxidation/reduction ability of transition-metal ions in the perovskite framework as a new modification approach. This material concept can be used to expand the semiconductive properties, as electron and hole doping can be freely tuned by controlling the charges of the layers. Additionally, precise fixation of the interlayer ion density could be utilized to apply layered perovskites in ionic material fields. A novel material concept for layered perovskites is based on the construction of a “charge-neutral layer” with a zero-valent perovskite layer. Many layered compounds with neutral layer charges, such as graphene,^11^ boron nitrides,^12^ transition-metal dichalcogenides (TMDs)^13^ and transition-metal carbides (MXenes),^14^ exhibit excellent performances as electronic and nanomaterials. However, 2D materials with charge-neutral layered perovskite units have not been reported until now. Poor redox activity species, such as Ti^4+^, Nb^5+^ and Ta^5+^, are often introduced at the B-site of most layered perovskites,^4,15,16^ while the A-site contains relatively large ions of alkaline earth metals and lanthanides that also show poor activity to redox. Even for layered perovskite compounds with a variety of chemical compositions, suitable parent materials containing redox active elements that can be used to realize novel material concepts have not been discovered. Thus, charge-neutral layered perovskites could be used to define a new material family.

Recently, we succeeded in discovering a new layered perovskite by introducing the trivalent cerium ion^17^ RbCeTa_2_O_7_ into a Dion–Jacobson-type layered perovskite structure denoted as M[A_*n−*1_B_*n*_O_3*n*+1_] (M: alkaline metals; n: number of octahedron sheets).^16^ This was the first report of a compound containing Ce ions in a layered perovskite capable of interlayer modification. The trivalent Ce ion exhibits the reversible redox performance of Ce(iii/iv) (*E* = 1.44 V *vs.* SHE).^18^ Trivalent Ce-containing compounds have been applied to automotive catalysis.^19^ The −1 charge ([Ce^III^Ta_2_O_7_]^−^) in the perovskite layer of the Dion–Jacobson-type RbCeTa_2_O_7_ could be neutralized to zero by the oxidation of trivalent Ce to realize a charge-neutral perovskite layer [Ce^IV^Ta_2_O_7_] through topotactic chemical reactions from the anionic [Ce^III^Ta_2_O_7_]^−^ layer as well as the occupation of the A-site of the perovskite structure by Ce^4+^. This charge-neutralization capability had never been reported throughout the long history of perovskite studies. The electronic structure of RbCeTa_2_O_7_ is completely different from that of RbLnTa_2_O_7_ (Ln = La, Pr, Nd and Sm), although both have similar ion-exchange abilities and structural features. The Ce in the perovskite lattice has one 4f electron due to the trivalent Ce; this electron exhibits metal-to-metal charge transfer (MMCT) transitions between Ce 4f and Ta 5d, forming the conduction band. Such MMCT transitions have rarely been observed in the conventional perovskite materials and complex oxides, indicating a unique electron transition. The electronic structure of a cerium-containing layered perovskite must be tuned to produce a unique electron transition through the control of the Ce oxidation state at the A-site and interlayer modifications.

In this work, we tested the preparation of a charge-neutral [Ce^IV^Ta_2_O_7_] layered perovskite by soft chemical oxidation of RbCeTa_2_O_7_ as a parent compound with a permanganate ion (MnO_4_^−^). The average and local structures of the obtained samples were investigated by X-ray diffraction and X-ray absorption spectroscopy, respectively, and the electronic structures were evaluated experimentally and theoretically. Furthermore, the chemical reactivity of the perovskite [Ce^III/IV^Ta_2_O_7_] layers was tested by XRD, XAFS and electrochemical analyses.

## Experimental section

### Materials

High-purity reagents were used as follows: TaCl_5_ (99.99%) was purchased from Kojundo Chem. Lab. Co., Ltd. (Japan); RbNO_3_ (99%), Ce(NO_3_)_3_·6H_2_O (99.9%), citric acid anhydrous (CA, C_6_H_8_O_7_; >98.0%), methanol (MeOH, CH_3_OH; >99.8%), ethylene glycol (EG, C_2_H_6_O_2_; >99.5%), ethylamine aq. solution (EA, C_2_H_5_NH_2_; 70%), tetrabutylammonium hydroxide aq. solution (TBAOH, [(*n*-C_4_H_9_)_4_N]OH; 10%), sulfuric acid (H_2_SO_4_; >95.0%) and KMnO_4_ (>99.3%) were purchased from Fujifilm Wako Pure Chem Co. (Japan); graphite powder and chloric acid (HCl; 35.0–37.0%) were purchased from Kanto Chemical Co., Inc.

### Synthesis of the RbCeTa_2_O_7_ parent material

The RbCeTa_2_O_7_ parent (Rb-form) was synthesized through the polymerized complex (PC) method according to the literature (see the detailed procedure in the ESI†).^17^ After the Rb-form (*ca.* 1.5 g) was added to 50 mL of 1 M HCl solution, the solution was stirred at room temperature for 4 days to produce HCeTa_2_O_7_·*n*H_2_O (H-form). The H-form was collected by filtration, washed with deionized water, and dried at 60 °C in a vacuum. For the intercalation reaction, the 1.0 g H-form compound was reacted with 7 wt% EA aqueous solution for 5 days at room temperature. The solution was centrifuged at 10 000 rpm to collect the EA-intercalated form sediment (EA-form). Then, 1.0 g of EA-form was reacted with 150 mL of 1 wt% TBAOH aqueous solution for 7 days at room temperature to induce delamination into the colloidal suspension containing the [Ce^III^Ta_2_O_7_]^−^ sheets. The spontaneous undelaminated precipitates formed after centrifugation at 2500 rpm for 20 min were removed by using the supernatant as a suspension. The suspension was centrifuged at 15 000 rpm for 15 min to obtain pale green delaminated [Ce^III^Ta_2_O_7_]^−^ powders (TBA-forms).

### Oxidation of the perovskite delaminated sheets

The charge-neutral [Ce^IV^Ta_2_O_7_] layered perovskite (Ox-form) was prepared through a soft chemical reaction at room temperature based on a chemical oxidation process using a KMnO_4_ oxidizing agent. The [Ce^III^Ta_2_O_7_]^−^ suspension (20 mL) was added dropwise to 20 mL of 0.2 M H_2_SO_4_ solution containing 2 mM KMnO_4_. The transparent purple solution then started to appear cloudy. The obtained suspension was centrifuged at 12 000 rpm to obtain the precipitants, which were washed with diluted H_2_SO_4_ solution and then deionized water more than 3 times until the filtrate became transparent. Finally, the collected pale-yellow powder was dried at 60 °C overnight in a vacuum.

### Chemical reactivity test

To confirm the oxidative capacity for the [Ce^IV^Ta_2_O_7_] perovskite layer, 20 mg of Ox-form sample was weighed and dispersed into 5 mL de-ionized water by sonication. Then, 5 mL of 10 mM l(+)-ascorbic acid was added into the [Ce^IV^Ta_2_O_7_] solution drop by drop with stirring at room temperature. After the [Ce^IV^Ta_2_O_7_] solution in the presence of l(+)-ascorbic acid was kept stirring for 10 min, the reacted powder was collected by centrifugation at 10 000 rpm for 10 min, washed with de-ionized water and dried at 60 °C *in vacuo*.

### Electrochemical measurements

For the electrochemical analysis, the TBA-form-deposited working electrode was fabricated as follows. The Au electrode with a diameter of 6 mm was firstly cleaned with acetone, EtOH and water by sonication for 20 min, respectively. After drying, the electrode was immersed in 2.5 wt% PEI solution for 20 min, and then the TBA-form suspension was added dropwise onto the PEI-coated Au electrode. After standing for 20 min, the TBA-form modified Au electrode was rinsed with water, and subsequently it was immersed in 20 wt% PDDA solution for 20 min. The PDDA/Ce^III^Ta_2_O_7_/PEI-Au electrode was finally washed using water and then dried in an ambient atmosphere.

Voltammetric measurements were carried out in acetonitrile containing 100 mM TBAPF_6_ used as the electrolyte with an electrochemical workstation (BAS 50W; Bioanalytical Systems, BAS). A standard three-electrode electrochemical system was utilized with the PDDA/Ce^III^Ta_2_O_7_/PEI-Au electrode, Ag and Pt wires as working, reference and counter electrodes, respectively. The raw potential measured using Ag reference electrode was calibrated by the ferrocene/ferrocenium ion (Fc/Fc^+^) scale using data derived from voltammograms for the oxidation of 1.0 mM Fc. The electrolyte was purged with Ar gas for more than 5 min. in advance to remove the dissolved oxygen in the solvent. The measurement was made at 25.0 ± 2 °C and the scan rate was set to 100 mV s^−1^.

### Material characterization

The crystal phase was determined by X-ray diffraction (XRD). The morphologies of the bulk samples and delaminated sheet were observed with electron microscopy techniques (scanning electron microscopy (SEM), transmission electron microscopy (TEM) and scanning transmission electron microscopy (STEM)) and atomic force microscopy (AFM), respectively. Ce and Ta L_3_-edge X-ray absorption fine structure (XAFS) and X-ray photoelectron spectroscopy (XPS) were employed to understand the chemical states and local structures of the obtained [CeTa_2_O_7_] derivative samples. The optical absorption properties of the samples were measured using an ultraviolet-visible-infrared (UV-vis-IR) spectrometer. The detailed characterization and theoretical calculation methods are also described in the ESI.†

## Results and discussion

### Chemical state analyses

**Fig. 1:**
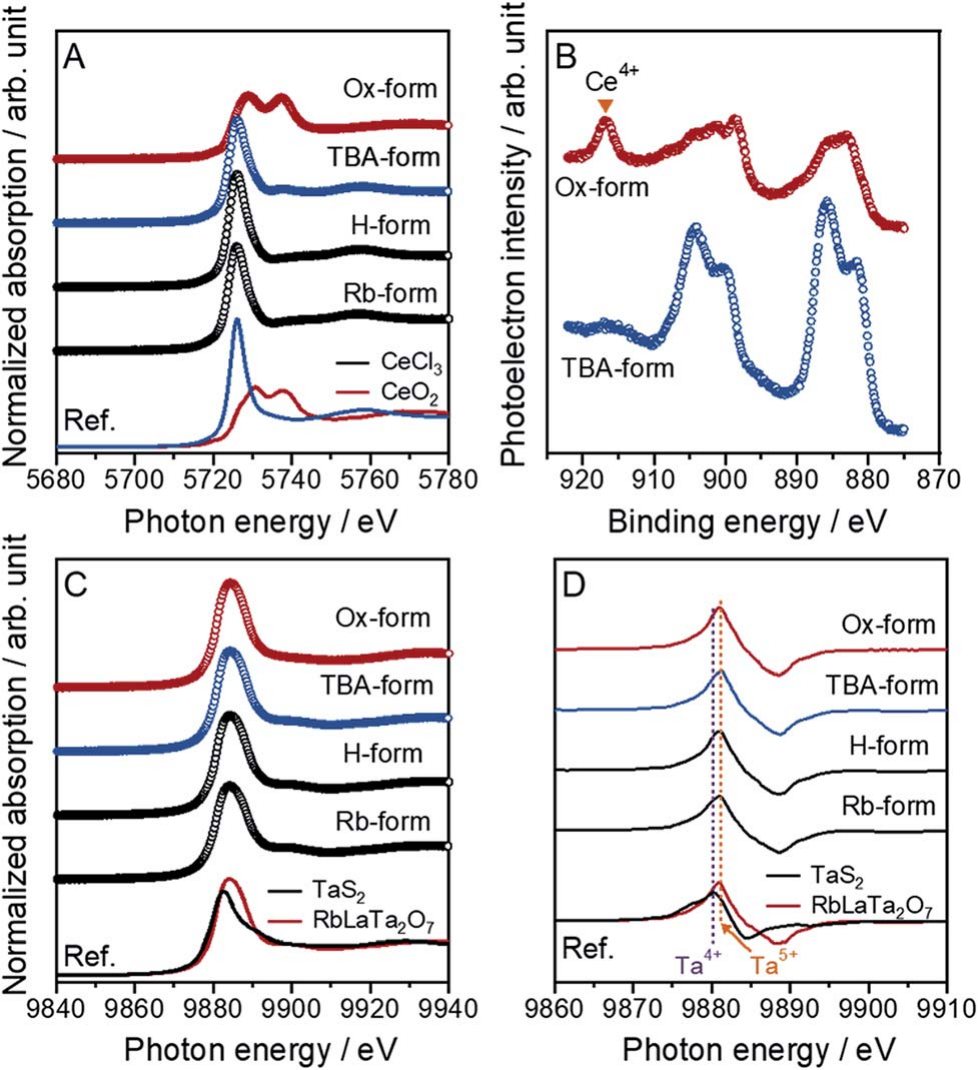
Normalized XANES spectra for the (A) Ce and (C) Ta L_3_-edges of the Ce-containing layered perovskite derivatives, Rb-, H-, TBA- and Ox-forms, with the reference compounds comprising the charged cations Ce^3+^ (CeCl_3_) and Ce^4+^ (CeO_2_) for the Ce L_3_-edge in (A) and Ta^4+^ (TaS_2_) and Ta^5+^ (RbLaTa_2_O_7_) for the Ta L_3_-edge in (C), respectively. (B) Ce 3d XPS spectra for the TBA- and Ox-forms. (D) The derivative Ta L_3_-edge XANES spectra for all forms and references.

It is difficult to control an atomic charge valence for cerium atoms in the crystal lattice because of the susceptibility to valence changes. However, this control is very important for the synthesis of Ce-based compounds. An accurate determination of the compound valence is also valuable. In this work, the oxidation states of Ce atoms for all forms of [Ce^III^Ta_2_O_7_]^−^ and [Ce^IV^Ta_2_O_7_] were precisely evaluated through an analysis of the X-ray absorption near edge structure (XANES) spectra for the Ce L_3_-edge (Fig. 1A). Specific XANES spectra of the Ce L_3_-edge, which are analogous spectra for the CeCl_3_ standard sample, were observed with a slightly asymmetric peak at 5726 eV for the Rb-, H- and TBA-forms, indicating that trivalent Ce (Ce^3+^) could be introduced into the main crystal lattice. Similar spectra were observed for the reported RbCeTa_2_O_7_ as well as other Ce-based compounds.^20,21^ The Ox-form exhibited two evident absorption peaks at 5729 and 5737 eV. The XANES spectrum of typical Ce^4+^-containing compounds such as CeO_2_ exhibits two peaks, as shown at the bottom of Fig. 1A, demonstrating that the Ce^4+^ ion should dominantly exist in the Ox-form. The quantitative analysis using CeCl_3_ and CeO_2_ as standard samples indicated that more than approximately 80% of Ce^3+^ or Ce^4+^ should be contained in the corresponding forms (Fig. S1†). The oxidation state of Ce on the particle surface was analyzed based on Ce 3d XPS spectra for all samples (Fig. 1B). Two main asymmetric bands appeared at approximately 880–890 and 895–910 eV in the TBA-forms, which were attributed to the 3d_3/2_ and 3d_5/2_ levels, respectively. Since such characteristic main bands are also observed in Ce^3+^-containing compounds such as CePO_4_,^22^ Ce must exist as a trivalent ion on the surface of the TBA-forms. In the case of the Ox-forms, a new band appeared at approximately 917 eV with two specific bands corresponding to Ce^3+^; these results are very similar to the characteristics of tetravalent Ce compounds such as CeO_2_.^22^ In all forms, the chemical states of Ce on the surface corresponded to the results of the XANES analysis.

This evidence indicated that the anionic layer [Ce^III^Ta_2_O_7_]^−^ was converted to a charge-neutral [Ce^IV^Ta_2_O_7_] layer through the oxidation of the Ce^3+^ ions in the TBA-form with MnO_4_^−^ ions to form Ce^4+^. The valence states of the tantalum in all forms were also confirmed to be pentavalent through the analysis of Ta L_3_-edge XANES spectra (Fig. 1C). The tantalum in the as-made RbLaTa_2_O_7_ was confirmed to exist in Ta^5+^ (Fig. S2†) based on the Ta L_3_-edge XANES spectra generated by utilizing Ta_2_O_5_ and TaS_2_ as the reference samples for Ta^5+^ and Ta^4+^, respectively. The same Ta L_3_-edge XANES spectra were obtained for all forms presented without any significant differences in the spectra. The average oxidation state in the mixed-valence compounds could be estimated from the peak position of the derivative XANES spectra, namely, the shift of the absorption edge.^23,24^ Generally, the absorption edge should shift to a higher energy as the oxidation state increases. Indeed, the derivative peak position of RbLaTa_2_O_7_ (Ta^5+^) was observed at higher energy than that of TaS_2_ (Ta^4+^) (Fig. 1D). On the basis of the relationship between the derivative peak positions and the oxidation states of transition metals, the oxidation states of tantalum in all forms should be pentavalent corresponding to the result of a similar absorption edge for RbLaTa_2_O_7_.

### Morphology and atomic arrangement observations

**Fig. 2:**
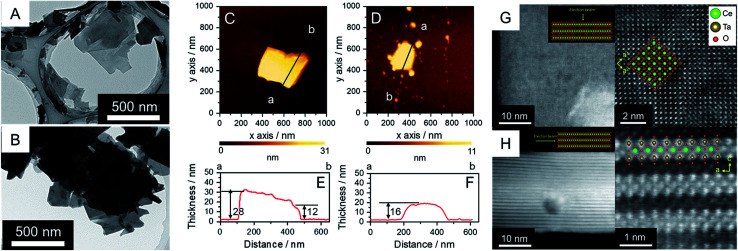
(A and B) TEM images and (C and D) AFM images with (E and F) thickness profiles for the (A, C and E) anionic TBA- and (B, D and F) charge-neutral Ox-forms. HAADF-STEM images of the Ox-form in the charge-neutral [Ce^IV^Ta_2_O_7_] from (G) basal and (H) vertical views for the layer; (left) lower and (right) higher magnifications.

RbCeTa_2_O_7,_ as a parent compound, has a plate-like morphology similar to the commonly found morphology in Dion–Jacobson-type layered perovskites.^25,26^ Similarly, based on the SEM observations reported previously, the synthesized Rb- and H-forms were determined to have plate particles (Fig. S3†) without remarkable size differences. On the other hand, the particles of the TBA- and Ox-forms decreased in size compared to those of the parent forms (Fig. S3†), although fewer differences in the particle morphologies in both samples were observed from the SEM images. The TEM images of the TBA-forms show that the particles were well dispersed (Fig. 2A and B) due to less interaction between the bulky TBA cation and the anionic layer. These images also show that the TBA-forms have plate-like morphologies with a lateral size of ∼500 nm, which is smaller than the parent Rb- and H-forms with a lateral size of ∼2 μm. In the case of the Ox-form, similar particle sizes and morphologies to the TBA-form were found, although the particles were agglutinated due to the strong van der Waals interactions in the charge-neutral layer. Because the TEM image cannot provide quantitative information on the thickness of the sheet particles, we utilized AFM images to determine the thickness details of the crystals (Fig. 2C–F). The thicknesses of the plates in the TBA- and Ox-forms were ∼30 and ∼20 nm, respectively. Since the theoretical thickness of the [CeTa_2_O_7_] perovskite monolayer was estimated to be ∼1 nm based on the crystal structure of RbCeTa_2_O_7_,^17^ the plate particle should have a laminated structure composed of 10–30 layers. The crystal thicknesses of the Rb- and H-forms were determined to be on the order of 100 nm based on SEM images (Fig. S3†). Thus, the TBA- and Ox-forms should be partially delaminated compared to the parent compound. Since the thickness of the exfoliated perovskite nanosheets with a single layer has been shown to be on the single nanometer scale, the [CeTa_2_O_7_] nanoplates in this work should be relatively bulky.

The atomic arrangement of the Ox-form particles was directly confirmed by high-angle annular dark-field (HAADF) scanning transmission electron microscopy (STEM) observations (Fig. 2G and H). Flat and standing particles were acquired for the sample holder STEM grid by carefully searching the sample to successfully observe the out-of-plane and in-plane atomic arrangements (Fig. S4†). The TEM and AFM images in Fig. 2A–F indicate that the nanoplate particles are ∼25 nm thick, which is in good agreement with the AFM image results. The atomic arrangement could be clearly observed in the out-of-plane direction because the incident electron beams can be sufficiently transmitted to the detector. The atomic arrangements observed in the TEM and AFM images were in good agreement with the atomic positions of the Ce and Ta heavy atoms in the crystal structure based on the perovskite structure in the [001] direction (Fig. 2G). On the other hand, the layered structure was evidently confirmed based on the STEM image of the in-plane direction, although the obtained STEM images were slightly blurred due to the insufficient transmission of the electron beam from the reduced crystallinity and increased depth of the particle (Fig. 2H). Atoms with a similar number of electrons, namely, Ce and Ta atoms, should exist throughout the layer because atomic shadows of similar brightness were observed both on the surface and at the center of the layer; however, it is difficult to distinguish the difference between Ce and Ta due to the ambiguous atomic boundary. In addition, the annular bright-field (ABF) STEM projected images had the opposite contrast of the HAADF-STEM image (Fig. S5†). Energy-dispersive X-ray spectroscopy (EDS) mappings provided evidence that the constituent elements of Ce, Ta and O were homogeneously dispersed over a single particle, but nonlinear column mapping indicated that Ce atoms certainly existed in the layer structure (Fig. S6†). Such HAADF-STEM images were also observed for the parent material of RbCeTa_2_O_7_,^17^ supporting the strong evidence regarding the presence of Ce atoms at the center of the perovskite layer.

### Crystal phase determination and its theoretical investigation

**Fig. 3:**
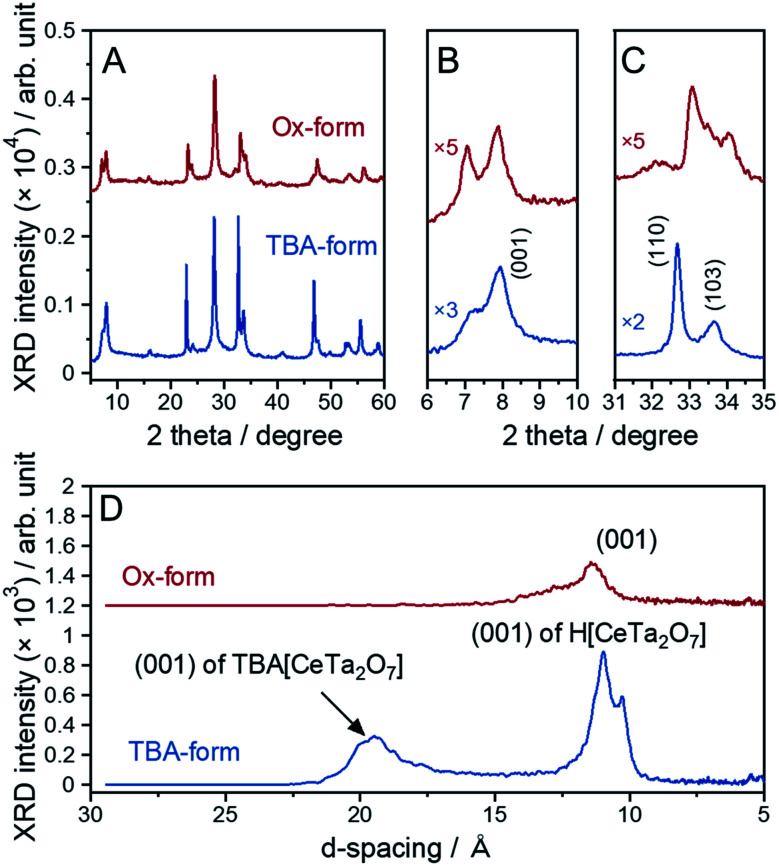
XRD patterns of the TBA- and Ox-forms: (A) overall pattern, (B) enlarged patterns with a range of 6–10° and (C) 31–35° and (D) lower-angle side.

The parent layered perovskite compound RbCeTa_2_O_7_ was discovered in our previous study.^17^ The crystal structure was determined to be in the space group of *P*4/*mmm* (No. 123) with the Dion–Jacobson type (Fig. S7†). The XRD pattern of the protonated form (H-form) is also shown in Fig. S8.† The XRD peaks of the H-form were broader than those of the Rb-form because of the lower ordering in the interlayer hydrogen bond arrangement found through ^1^H magic-angle spinning nuclear magnetic resonance (MAS NMR) measurements (Fig. S8†). In a tetragonal structure, the lattice constants for each crystal axis can be determined based on the *d*-spacing changes in [110] for the *a*- and *b*-axes and [001] for the *c*-axis. The lattice constants for the H-form calculated from the XRD pattern were estimated to be *a* = 3.8766(2) and *c* = 10.785(2) Å, which are slightly shorter than those of the La analog (HLaTa_2_O_7_; *a* = 3.885(2) and *c* = 11.106(6) Å)^27^ due to the difference in the ionic radius (La^3+^: 1.16 Å; Ce^3+^: 1.143 Å, for 8-fold).^28^ Many protonated Dion–Jacobson-type layered perovskites easily accept water molecules into the interlayer under acidic conditions.^26,29,30^ The H-form sample in this work was also compatible with such water molecule introduction. The amount of water (H_2_O) was determined to be approximately 0.5, denoted as HCeTa_2_O_7_·0.5H_2_O, based on thermogravimetric (TG) analysis (Fig. S9A†). In the H-form reacted with EA, a new XRD peak appeared at a lower angle than the (001) diffraction peak of the H-form; thus, EA was shown to intercalate into the interlayer of HCeTa_2_O_7_ (Fig. S7†). A similar intercalation reaction occurs in the La analog of HLaTa_2_O_7_ through an acid–base reaction during the nanosheet synthesis process.^31^ By reacting the H-forms with a bulky organic cation such as a tetrabutylammonium ion, (*n*-C_4_H_9_)_4_N^+^ (TBA^+^), the perovskite stacks could be stretched and exfoliated to form a sheet morphology at the nano-order level.^31^ The fully exfoliated monolayer nanosheets produce strong XRD peaks from (00*l*) stacking planes.^32^ The XRD profile of the prepared TBA-form was similar to those of Dion–Jacobson structure types HCeTa_2_O_7_ and RbCeTa_2_O_7_, implying that exfoliation did not fully occur and laminations with several layers remained in the sample (Fig. 3A–C). However, the TBA^+^ cation could also be introduced into the interlayers, and the structures were partially exfoliated to form monolayer nanosheets. This result was determined because a strongly sharp XRD diffraction peak was observed due to the stacking direction of the (00*l*) plane and the other XRD peaks related to the in-plane directions were broadened. In addition, an XRD peak with a large *d*-spacing (*ca.* 19 Å) for the TBA-form was observed at 4.65° in the low-angle XRD pattern (Fig. 3D). The XRD patterns of the Ox-form were quite similar to those of the TBA-form, whereas interestingly, anisotropic diffraction peak shifts were observed in the [Ce^IV^Ta_2_O_7_] due to the oxidation of Ce^3+^. Particularly, the XRD peak position of the in-plane direction (*hk*0) on the *a*- and *b*-axes shifted to the higher angle side, while no evident peak shift was observed for the stacking (00*l*) plane on the *c*-axis. Therefore, the anisotropic lattice change indicates the occurrence of the chemical oxidation of the anionic [Ce^III^Ta_2_O_7_]^−^ to charge-neutral [Ce^IV^Ta_2_O_7_] layers. In addition, some XRD peaks of the Ox-form were broadened and asymmetric, which may be due to the decrease of crystallinity with the chemical treatment as well as the existence of the chemical species, *e.g.* H_2_O, in the interlayer.

**Fig. 4:**
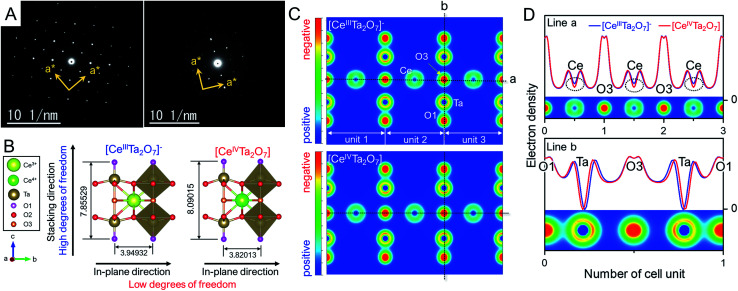
(A) SAED patterns of the (left) anionic TBA- and (right) charge-neutral Ox-forms. (B) Schematic structures of the anionic [Ce^III^Ta_2_O_7_]^−^ and charge-neutral [Ce^IV^Ta_2_O_7_] perovskite layers optimized based on DFT calculations (the bond length unit is Å). (C) Simulated electron density distributions on the (110) plane and (D) electron density profiles at lines a and b in F for the anionic [Ce^III^Ta_2_O_7_]^−^ and charge-neutral [Ce^IV^Ta_2_O_7_] perovskite sheets.

Almost the same selected-area electron diffraction (SAED) patterns of the TBA- and Ox-forms were observed to have clear spot patterns (Fig. 4A), indicating that the plate particles of both forms are individual single crystals. In addition, both SAED patterns were quite similar to the simulation pattern of the [001] direction in the parent RbCeTa_2_O_7_ (Fig. S10†), as the lateral plane of the plate particle was oriented in the direction of the {100} planes. In the two-layer Dion–Jacobson type layered perovskites, such as [LnM_2_O_7_] layers (Ln: lanthanides; M: Ta and Nb), it is well known that the B-site octahedron tilts by introducing lanthanide ions with different sizes.^33^ For example, RbNdTa_2_O_7_ layered perovskites can change the octahedron tilting depending on the temperatures.^34^ At room temperature, it can take the alternated stacking structure with the tilting polar perovskite layers of the space group of *I*2cm, which can be presented by *a*^−^*a*^−^c^+^/−(*a*^−^*a*^−^*c*^+^) tilting as the Glazer notation,^35^ whereas, as the temperature increases, the structure take an anti-polar (*Cmca*), non-polar (*I*4/*mcm*) and then finally a non-tilting *P*4/*mmm* phase. Such layered perovskite with octahedral tilting takes the supercell of 2*a* × 2*a* or 
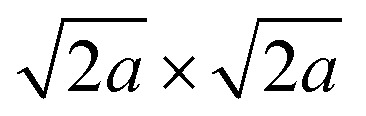
 for viewing from *c*-axis; however, a displacement can be observed due to the tilting of the oxygen ligand around the B atoms between two layers. Thus, the presence/absence of the octahedron tilting can be determined by SAED pattern analysis because if the material has some tilting, *i.e.* symmetry is lower, the diffraction spot corresponding to the displacement of the oxygen should be observed. Therefore, the SAED patterns of the Ox-form were carefully analyzed by adjusting the contrast and brightness; however, we could not confirm even the ultraweak reflections originating from the octahedral tilting. The result of the fine analysis of SAED patterns provides evidence that the bright spots in Fig. 4A is only (001) direction as well as the structure does not have octahedral tilting, meaning that it has tetrahedral symmetry. As is known from the estimation of the stability of the perovskite using the tolerance factors, in general, the tilting is affected by the ratio of the ionic radius of A- and B-site cations. The ionic radius of Ce^4+^ (1.14 Å, CN = 12)^28^ is smaller than that of Ce^3+^ (1.34 Å, CN = 12)^28^ as well as smaller than that of Nd^3+^ (1.27 Å, CN = 12)^28^ ions; thus, the Ox-form should take the more significant tilting than the RbNdTa_2_O_7_ from the viewpoint of solid-state chemistry. However, the result of the SAED analysis indicated no tilting in the Ox-form, which is quite irregular for the perovskite material, suggesting that the charge neutral [CeTa_2_O_7_] layer synthesized in this study has some special atomic arrangement.

We presumed that the irregularity of such tetragonal structure with no-tilting originated from the anisotropic lattice change from the TBA- to the Ox-form as mentioned in the XRD analysis section. To reveal the anisotropic lattice changes in [Ce^IV^Ta_2_O_7_] indicated by XRD results, theoretical calculations based on density functional theory (DFT) were carried out for both anionic [Ce^III^Ta_2_O_7_]^−^ and charge-neutral [Ce^IV^Ta_2_O_7_] forms. Perovskite mono-layers with vacuum layers of 12 Å were employed as structural models to simplify the calculation, unlike the multilayer perovskite particles in the real sample, and the perovskite unit of RbCeTa_2_O_7_ was utilized for the initial structure. Through lattice relaxation optimization, the crystal structure was maintained as layered perovskite units (Fig. 4B, which is illustrated by using the VESTA program^36^). The optimized lattice parameters and bond lengths of the anionic [Ce^III^Ta_2_O_7_]^−^ and charge-neutral [Ce^IV^Ta_2_O_7_] perovskites from the calculation by fixing the alternative symmetry of the *P*4*mm* (no. 99) space group are summarized in Table 1. The incorporation of smaller ions into the same crystal lattice generally shrinks the whole lattice, depending on the introduced ion size, according to Vegard's rule.^37^ Since the Ce^4+^ (1.14 *Å*, CN = 12)^28^ in [Ce^IV^Ta_2_O_7_] is smaller than the Ce^3+^ (1.34 Å, CN = 12)^28^ in [Ce^III^Ta_2_O_7_]^−^, the lattice constants for the in-plane directions of the *a*- and *b*-axes shrank −3.27%, whereas that in the stacking direction of the *c*-axis clearly expanded *ca.* +2.99%. This calculation result is consistent with the experimental XRD analysis results. The anisotropic lattice shrinkage/expansion for charge-neutral [Ce^IV^Ta_2_O_7_] can be mainly explained by the geometric change in the TaO_6_ octahedron in the perovskite framework. Such changes are quantified according to the differences in three types of Ta–O bond lengths between apical (O1), equatorial (O2) and bridging oxygens (O3) and centered Ta ions. Compared with the DFT calculation, the bond length of Ta–O2 in the in-plane direction was 0.94% shorter than that of [Ce^III^Ta_2_O_7_]^−^; this difference is related to the change in the lattice parameter. In contrast, in the stacking direction, the bond length of Ta–O1 shrank −1.92%, while the length of Ta–O3 expanded by +7.00%. On the other hand, two types of Ce–O bonds that form CeO_12_ icosahedrons shrank 2.22% for Ce–O2 and 3.27% for Ce–O3, respectively. The distance between Ce and Ta was also slightly separated by 0.71%. The relative position of the Ta ion in the perovskite framework was displaced toward the apical direction, resulting in a longer bond length for Ta–O3. On the basis of the above bonding change results, the anisotropic lattice change should be attributed to the following reasons: shrinkage in the in-plane direction due to a decrease in the ionic radius of Ce generated *via* Ce oxidation from +III to + IV and expansion in the stacking direction due to an increase in the bond length of Ta–O3 (Fig. 4B and Table 1). This irregular expansion toward the stacking direction irrespective of the ionic radius order can be understood on the basis of electrostatic interactions between the cations in the anionic/charge-neutral layer frameworks. The electron density maps of each perovskite were simulated through DFT calculations (Fig. S11† and 4C). Although no apparent differences can be observed from the 2D electron density distributions in the (110) plane, differences in the electron density between Ce and Ta were clearly observed from the corresponding line profiles (Fig. 4D). There was no clear difference in the electron density of the Ta atom in the two perovskite layers, while that of Ce in the charge-neutral [Ce^IV^Ta_2_O_7_] layer exhibited a noticeable positive shift, namely, a positive charge bias, indicating that the electrostatic repulsion between the Ce and Ta atoms should increase with an increasing positive shift of the Ce atom. The B-site (Ta atom) in the 2D perovskite layer had fewer degrees of freedom in the in-plane direction because of crystallographic constraints, but it had more degrees of freedom in the stacking direction due to the lack of crystallographic constraints. Thus, the Ta atom significantly shifted in the direction of the layer surface through Ce–Ta electrostatic repulsion, resulting in the expansion of the bond length of Ta–O3. On the other hand, the Ce atoms formed strong bonds with surrounding oxygens, and the Ce–O bond lengths became isotopically short. Therefore, the anisotropic lattice change based on chemical oxidation was concluded as follows: the shrinkage in the in-plane direction originated from the contraction of the ionic radius of Ce and electrostatic attractive forces between Ce and O, and the expansion of the stacking direction was attributed to the increase in the electrostatic repulsion between Ce and Ta. Indeed, the centered metals in the octahedron, namely, B-site atoms, can be displaced toward the stacking directions by changing the charge of the layers in layered perovskite compounds.^38^ For example, in Ruddlesden–Popper-type Li_*x*_LaTa_2_O_7_, the charges of the perovskite layer are equal to −1 for *x* = 1 and −2 for *x* = 2. The Ta–O3 bond length (2.262(6) Å) in LiLaTa_2_O_7_ is *ca*. 5% longer than that in Li_2_LaTa_2_O_7_ (2.154(3) Å).^38^ In other words, the displacement of the B-site should depend on the sum of the ion charge at the A- and B-sites, namely, the magnitude of the electrostatic repulsions. In general, it is well known that the introduction of smaller ions into the A site leads to tilting of the B-site octahedron. However, although the smaller ion of Ce^4+^ (1.14 Å; CN = 12) was introduced into the A-site of the perovskite lattice, it was formed as the tetragonal structure, which is irregular from the viewpoint of solid-state chemistry. It can be suggested that the flexibility due to the stacking direction shift of the Ta position promotes the stabilization of the tetragonal structure. Additionally, the delamination of the [CeTa_2_O_7_] perovskite layer should accelerate the displacement of Ta to contribute to the stabilization of the tetragonal structure, *i.e.* relaxing the tilting. This unique feature should be realized in other layered perovskites; however, it might be necessary to devise the chemical compositions that increase the positive repulsion between A- and B-sites.

**Table 1:** Lattice parameters of *a* (=*b*), thicknesses of the perovskite monolayer and atomic distances of the anionic [Ce^III^Ta_2_O_7_]^−^ and charge-neutral [Ce^III^Ta_2_O_7_] perovskite layers optimized from DFT calculations

	[Ce^III^Ta_2_O_7_]^−^ (Å)	[Ce^IV^Ta_2_O_7_] (Å)	Differences (%)
*a* (=*b*) axis	3.94932	3.82032	−3.27
Thickness^a^	7.85529	8.09015	+2.99
Ce–O2 ×8	2.62275	2.56451	−2.22
Ce–O3 ×4	2.79259	2.70137	−3.27
Ta–O1	1.76286	1.72898	−1.92
Ta–O2 ×4	2.02279	2.00371	−0.94
Ta–O3	2.16476	2.31623	+7.00
Ce–Ta	3.53339	3.55841	+0.71

a Thickness of the perovskite monolayer and O1–O1 atomic distance.

### Local structure determination by EXAFS analysis

**Fig. 5:**
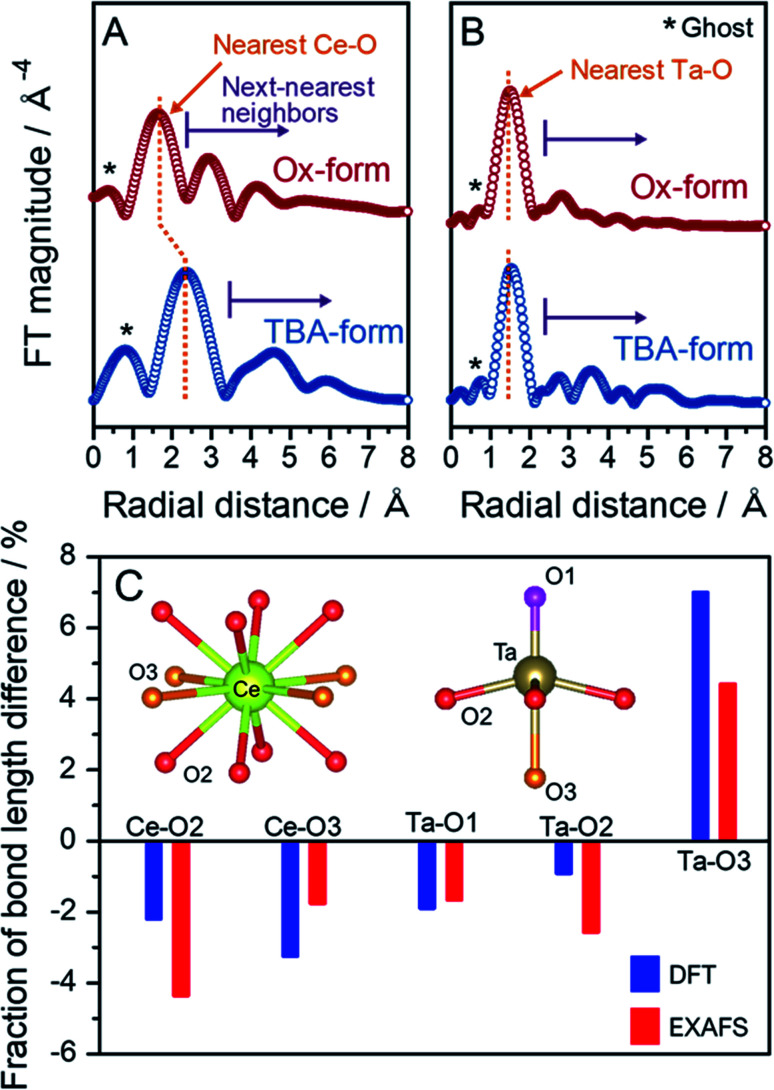
Fourier transformed (FT) EXAFS spectra in the *R*-space at the (A) Ce and (B) Ta L_3_-edges for the TBA- and Ox-forms. (C) Percentages of the bond length changes between the TBA- and Ox-forms obtained from DFT calculations and EXAFS analysis.

To support the validity of the structural optimization through DFT calculations and determine the local structures of Ce and Ta, EXAFS analysis based on the crystal structure optimized by DFT calculations was carried out. This method was chosen because the TBA- and Ox-forms do not have sufficiently high crystallinities to identify the structures from the XRD results. The EXAFS vibrations for each edge extracted from the overall XAFS spectra were weighted to the third power with the *k*^3^-function (Fig. S12†). The different *k*^3^-weighted EXAFS spectra in the TBA- and Ox-forms for the Ce L_3_-edge suggested that there was a significant change in the coordination conformation around Ce. No clear differences were observed in the Ta L_3_-edge spectra, although slight differences were observed at higher wavenumbers. The bond length details for Ce–O and Ta–O were estimated with the Artemis program using the Ce and Ta L_3_-edge XAFS spectra. The EXAFS analysis was conducted with the inverse-transform EXAFS spectra, so-called *q*-spacing (Fig. S13†). The Ce and Ta L_3_-edges Fourier transformed (FT) EXAFS spectra for the *R*-space for the TBA- and Ox-forms are shown in Fig. 5A and B, respectively. In the Ce L_3_-edge, one major peak was observed at a radial distance of 2–3 Å for each form, and some smaller peaks were observed at larger distances. Since the main peak of the nearest-neighbor bonding Ce–O appeared at approximately 2 Å in CeO_2_,^39^ the major peak at 2–3 Å and minor peaks should be assigned to the nearest Ce–O bond and the next-nearest-neighbor bonding species such as Ce–Ce, Ce–Ta and Ce–O. Since the long-distance information in the Ce L_3_-edge XAFS spectrum is susceptible to other L_2_-edge absorptions, we investigated only the nearest peak to quantitatively refine the EXAFS spectra. The local structure surrounding the Ce in the TBA-form was not different from the local structures in the Rb- and H-forms (Fig. S14†). The FT-EXAFS spectrum for the Ox-form exhibited a nearest-neighbor Ce–O peak shift towards the shorter distance and a significant change in the next-nearest-neighbor region compared with that of the TBA-form; these results indicated that the bond lengths around Ce in the Ox-form were shortened significantly. Dion–Jacobson-type double-layered perovskites such as RbCeTa_2_O_7_ have two oxygen atoms with different environments coordinated that are with the central A-site, *i.e.*, the Ce^3+^ ion is coordinated with two oxygen sites to form 12-fold A-sites. The Ce–O2 and Ce–O3 bond lengths were estimated to be 2.646(4) and 2.749(6) Å for the TBA-form and 2.53(1) and 2.70(5) Å for the Ox-form, respectively (Table 2), as well as the all fitting parameters were summarized in Table S1.† This shortening of the bond distance was similar to the corresponding result in the DFT calculation. The Ce–O bond length in CeO_2_ is estimated to be 2.35(2) Å based on EXAFS fitting,^40^ a value that is reasonably similar to the Ce–O bond length in the Ox-form. A ghost peak and the nearest Ta–O major peak were observed at ∼1 and approximately 1.5 Å, respectively, for the Ta L_3_-edge EXAFS spectra in each form (Fig. 5B). The peak position due to the nearest Ta–O in the Ox-form slightly shifted in the shorter bonding direction more than that in the TBA-form. The next-nearest-neighbor peaks slightly changed through the shortening of the nearest Ta–O bond distance; this shortening occurred due to the contraction of the entire lattice associated with the shrinkage of the Ce–O bonds and the disturbance of the long-range order. The quantitatively analyzed local Ta–O bonds are also summarized in Table 2. The fractional change in the bond lengths of Ce–O and Ta–O between the TBA- and Ox-forms was obtained based on DFT calculation and EXAFS analysis (Fig. 5C). The three types of Ta–O bond lengths that were experimentally determined from the EXAFS measurements were in good agreement with those obtained from the theoretical calculation. In particular, the experimentally determined Ta–O3 bond length was almost the same as the bond length determined based on the structure optimized in the DFT calculations. Therefore, the local structures of Ce and Ta obtained from the quantitative EXAFS analysis were validated based on DFT calculations for the TBA- and Ox-forms. In the summary of the results of DFT calculations and EXAFS analyses, the *c*-axis displacement of Ta atoms can work to relax the octahedral tilting, which would otherwise occur, to take a high symmetric tetragonal structure with no tilting. This new knowledge is one of the notable findings in this work because it is not based on conventional solid-state chemistry and is expected to further develop perovskite structures.

**Table 2:** Bond lengths, coordination numbers, and Debye–Waller factors obtained through first-shell EXAFS fitting at the Ce and Ta L_3_-edges in the TBA- and Ox-forms^a^

Derivatives	Path	*N*	*R* (Å)	*σ* ^2^	Path	*N*	*R* (Å)	*σ* ^2^
TBA-form	Ce–O2	8	2.646(4)	0.0071(7)	Ta–O1	1	1.78(3)	0.012(6)
	Ce–O3	4	2.749(6)	0.0001(9)	Ta–O2	4	2.002(6)	0.009(1)
					Ta–O3	1	2.26(3)	0.007(5)
Ox-form	Ce–O2	8	2.53(1)	0.012(3)	Ta–O1	1	1.75(8)	0.01(2)
	Ce–O3	4	2.70(5)	0.017(8)	Ta–O2	4	1.95(1)	0.008(2)
					Ta–O3	1	2.36(10)	0.01(2)

a *R*: bond length; *N*: coordination number; *σ*: Debye–Waller factor; parenthesis: standard deviation.

### Optical absorption properties

**Fig. 6:**
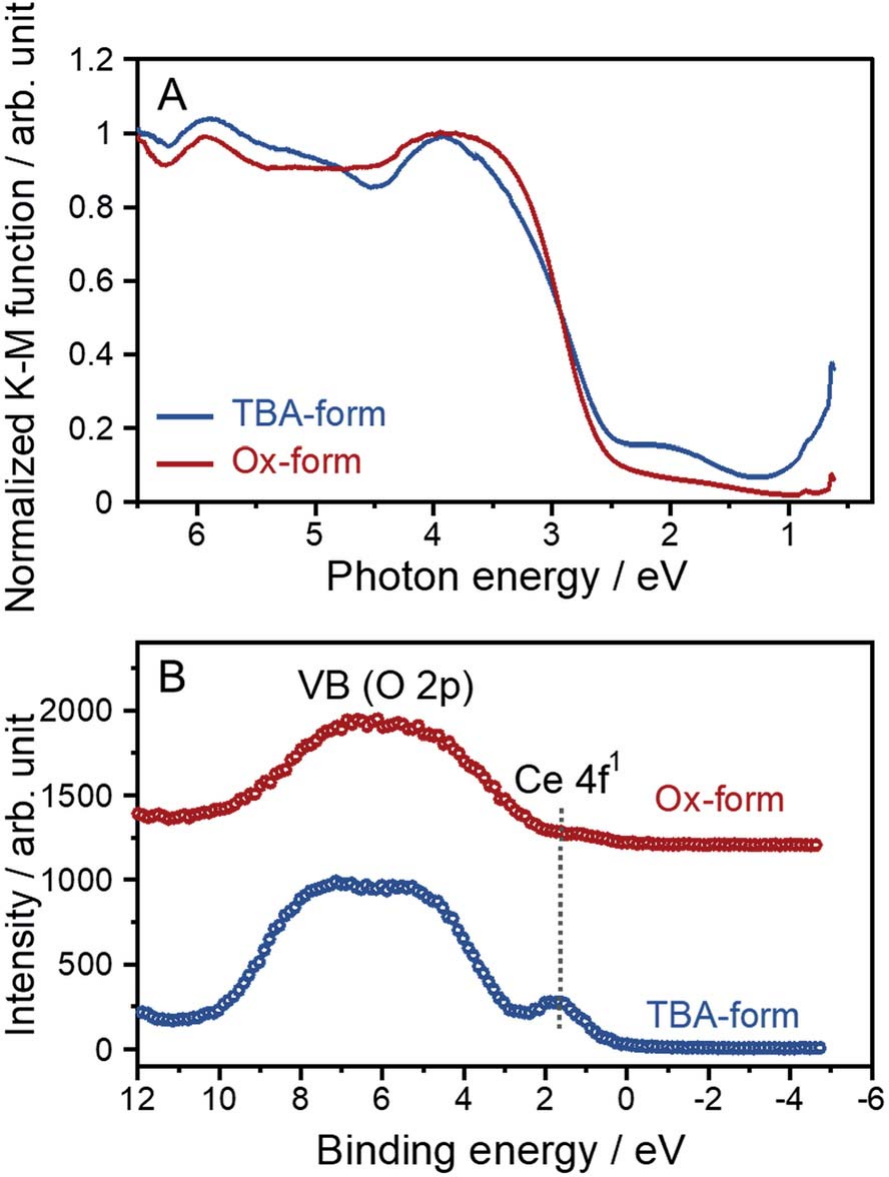
(A) Tauc plots and (B) VB XPS spectra for the TBA- and Ox-forms.

A cerium-introduced layered perovskite has unique optical absorption properties based on the location of the Ce 4f orbital in the bandgap at the mid-state level.^17^ In particular, RbCeTa_2_O_7_ powder exhibits a specific pale green color due to the two absorption types due to different electron transitions that occur in the blue regions; these transitions include the ligand-to-metal charge transfer (LMCT) transition from the O 2p (valence band) to the Ce 4f mid-state and the near infrared (NIR) regions through the MMCT transition from the Ce 4f mid-state to Ta 5d (conduction band). No reports have indicated that such multiple transitions, including the MMCT transition, occur among any well-known layered perovskites except for RbCeTa_2_O_7_. Therefore, we measured the diffuse reflectance (DR) spectra in the UV-visible-NIR region for the TBA- and Ox-forms with a Tauc plot (Fig. 6A). The specific NIR absorption was observed at approximately 1–2 eV in the TBA-forms, which is similar to the DR spectrum of RbCeTa_2_O_7_. However, the NIR absorption in the Ox-form decreased in intensity. To visualize the decrease in NIR absorption, valence band (VB) XPS spectra were observed, as shown in Fig. 6B. In the TBA-form, due to the Ce 4f level, an evident peak was observed at approximately 2 eV with a broad shoulder band due to the O 2p level at 3–10 eV. This shoulder band forms a valence band, which is similar to that in the RbCeTa_2_O_7_ parent material, while the corresponding Ce 4f peak disappeared in the Ox-form. Therefore, the decrease in NIR absorption should be ascribed to the lack of the mid-state Ce 4f electrons located at the near-Fermi level. The energy gaps of the LMCT and MMCT transitions were estimated to be 2.47 and 1.40 eV for the TBA-form and 2.54 and 1.12 eV for the Ox-form, respectively. Additionally, the optical absorption differences in the chemical oxidation from [Ce^III^Ta_2_O_7_]^−^ to [Ce^IV^Ta_2_O_7_] induced drastic coloring changes from pale green to pale yellow. To quantitatively compare the hue changes in both forms, the chromatic properties were investigated in terms of the Commission Internationale de l'Éclairage (CIE) L*a*b* system based on the DR spectra, in which each L*a*b* value is summarized in Table S2.† The chromatic values of the Ox-form increased compared to those of the TBA-form; in particular, the b* value drastically changed from 11.84 to 18.65, which indicates an increase in the yellow color composition and a decrease in the blue color composition.

### Electronic structure calculation

**Fig. 7:**
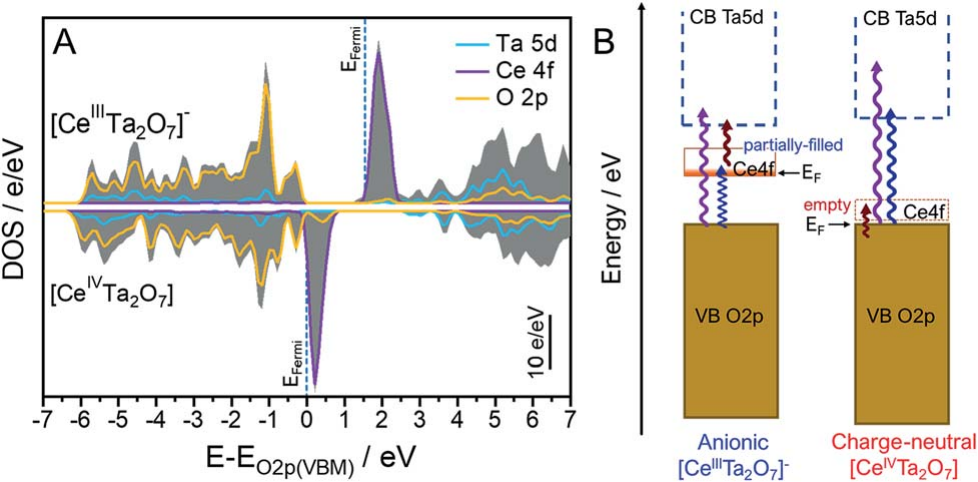
(A) Total (gray solid area) and partial DOSs (lines) for the Ce 4f (pink), Ta 5d (cyan) and O 2p (yellow) orbitals in the (top) anionic [Ce^III^Ta_2_O_7_]^−^ and (bottom) charge-neutral [Ce^IV^Ta_2_O_7_] perovskite layers. The VBM of the [Ce^III^Ta_2_O_7_]^−^ layer was drawn by shifting the peak top of the O 2p occupied orbital to be equal to that of [Ce^IV^Ta_2_O_7_] layers. (B) Schematic band diagram comparison for the anionic [Ce^III^Ta_2_O_7_]^−^ and charge-neutral [Ce^IV^Ta_2_O_7_] perovskite layers.

The total and partial density of states (DOS) plots for each [Ce^III^Ta_2_O_7_]^−^ and [Ce^IV^Ta_2_O_7_] layered perovskite were calculated from the same optimized structure with the DFT method to obtain a reasonable understanding of the optical absorption changes after chemical oxidation from [Ce^III^Ta_2_O_7_]^−^ to [Ce^IV^Ta_2_O_7_] (Fig. 7A). The VB and the conduction band (CB) for the [Ce^III^Ta_2_O_7_]^−^ layer were mainly formed from the O 2p bonding orbitals and from Ta 5d empty orbitals that were partially hybridized with the O 2p antibonding orbital, respectively; they are not related to the Ce 4f orbitals. Moreover, the Ce 4f state was located at the interband between the VB and CB as the mid-state. Each of the band positions of the VB, CB and mid-state in the [Ce^IV^Ta_2_O_7_] layer was similarly due to the O 2p, Ta 5d and Ce 4f levels, respectively. Although the energy gap between the VB maximum (VBM) and CB minimum (CBM) could be found to be similar in the [Ce^III^Ta_2_O_7_]^−^ and [Ce^IV^Ta_2_O_7_] layers, the Ce 4f orbital levels were observed in the different positions, which is a lower energy shift with the disappearance of the Ce 4f electron due to oxidation. The Fermi level in the [Ce^IV^Ta_2_O_7_] layer was formed from the O 2p orbitals in conjunction with the CB, meaning that the 4f electrons are not fully occupied in the Ce 4f level. The empty Ce 4f level was located at a ∼1 eV higher energy potential than the VBM (Fermi level). The position of the Ce 4f level in the [Ce^IV^Ta_2_O_7_] layer is lower than the Ce^4+^-doped zirconates and cerates, which has the empty Ce 4f levels at ∼2 eV higher than the VBM.^41–43^ Since the Ta 5d orbital forming CB is lower than that of Zr 4d, the Ce 4f orbital position may be decreased by its repulsion. Based on these DOS results, a schematic diagram of each band position and the electron-transition mechanisms of the two forms is provided in Fig. 7B. The [Ce^III^Ta_2_O_7_]^−^ layer exhibits multiple absorptions from two types of LMCT transitions: from the VB to the CB and then the VB to Ce 4f and from one MMCT transition and then Ce 4f to the CB, which results in NIR absorption. In contrast, the [Ce^IV^Ta_2_O_7_] layer exhibited absorption from only two electronic transitions of LMCT transitions without the MMCT transition because of the empty Ce 4f level. Therefore, the [Ce^IV^Ta_2_O_7_] layer absorbed only two regions of UV and blue light with weak NIR absorption and was colored pale yellow.

### Chemical reactivity tests

**Fig. 8:**
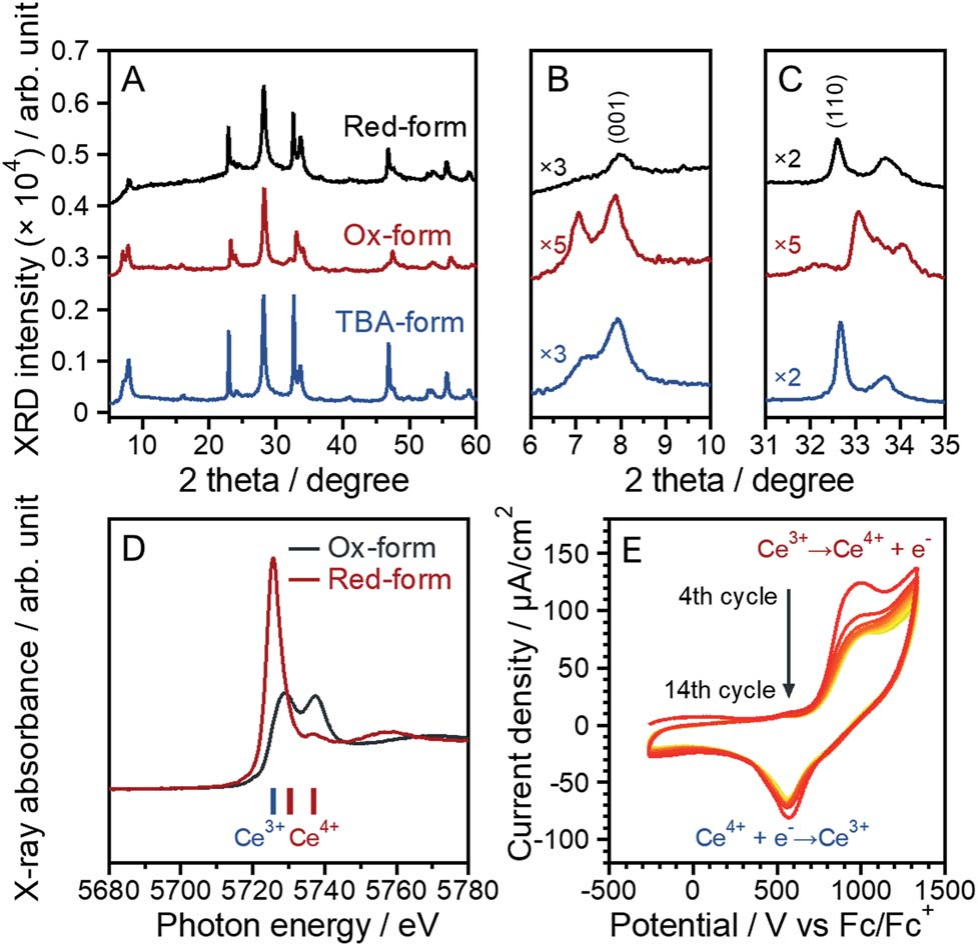
(A-C) XRD patterns of the TBA-, Ox- and red-forms. (D) Ce L_3_-edge XANES spectra of the Ox- and red-forms. (E) Cyclic voltammograms of the fabricated electrode at a scan rate of 100 mV s^−1^. The CV measurement was conducted in acetonitrile containing 100 mM TBAPF_6_.

The tetravalent Ce ion, Ce^4+^, containing inorganic compounds, typically CeO_2_ and (NH_4_)_2_[Ce(NO_3_)_6_] (CAN), exhibit excellent oxidative performance.^19,44^ Similar oxidative capacity was expected to be found in this unique perovskite layer with Ce^4+^ of [Ce^IV^Ta_2_O_7_]. Firstly, the reduction behavior of Ce^4+^ with ascorbic acid was investigated to evaluate the oxidic reactivity of the [Ce^IV^Ta_2_O_7_] perovskite layer. The XRD pattern of the red-form shows no obvious change from before the reduction test (Fig. 8A–C), indicating that the layered perovskite unit was retained. Furthermore, the slight expansion and shrinkage of the lattice for the red-form could be observed in the in-plane [110] and stacking [001] directions, respectively. This phenomenon is an opposite trend compared with the change in the lattice due to the oxidation reaction of the TBA-form to the Ox-form. Two X-ray absorption peaks at 5729 and 5737 eV were observed in the Ce L_3_-edge XANES spectrum of the Ox-form (Fig. 8D), whereas the red-form also shows a tiny peak at 5737 eV due to Ce^4+^ with a dominant peak at 5725 eV in which it was almost same peak position in the Ce L_3_-edge XANES spectrum of the parent forms of Rb-, H- and TBA-forms (5726 eV). In the multilayered perovskite, the reduction does not proceed sufficiently, and a part of Ce in the perovskite unit could not be reduced, resulting that it is supposed that the Ce^4+^ state was kept. These results of the reductive reduction tests for the [Ce^IV^Ta_2_O_7_] perovskite layer could indicate that the [Ce^IV^Ta_2_O_7_] perovskite layer can be reversibly reduced to the original [Ce^III^Ta_2_O_7_]^−^ anionic layer. The oxidative capacity of the [Ce^IV^Ta_2_O_7_] perovskite layer was demonstrated by voltammetric behavior in acetonitrile. Although the cyclic voltammogram of the fabricated PDDA/Ce^III^Ta_2_O_7_/PEI-Au electrode in the first cycle showed several undefined waves, a couple of waves were observed in the second and third cycles (Fig. S16A†). The cyclic voltammograms of the fabricated PDDA/Ce^III^Ta_2_O_7_/PEI-Au electrode in the 4th to 14th cycles are shown in Fig. 8E. The oxidation wave appeared at around 1000 mV, and the corresponding reduction wave at around 600 mV. Since these waves were not observed in similar measurements using bare Au and PEI-coated Au electrodes (Fig. S16B†), they are originated from the redox of the [CeTa_2_O_7_] perovskite layers. It is well known that the reduction potential of Ce^4+^ is *a* +1.44 V *vs.* SHE.^18^ The reduction potential of [Ce^IV^Ta_2_O_7_] perovskite layers can be converted to be +1300 mV (+1.3 V) *vs.* SHE from the redox potential of Fc/Fc^+^*vs.* SHE (+0.7 V), which is comparable to the standard potential of Ce^4+^. These redox waves should correspond to the redox reaction of Ce inside the perovskite unit. This electrochemical redox reaction is chemically reversible because a couple of waves were observed for at least 14 potential cycles. However, the magnitude of waves decreased in current as the potential cycle proceeded. This may be attributed to the fact that the reductive reaction is not completely proceeded due to small amount of Ce^4+^ residue, as evidenced by the above XANES result of the reduction behavior using ascorbic acid. To the best of our knowledge, the electrochemical behavior of layered perovskite materials *via* charge-neutral layers has been investigated for the first time.

## Conclusion

In this work, we first proved the existence of a layered perovskite tantalate containing tetravalent cerium (Ce^4+^) at the A-site through experimental and theoretical approaches based on DFT calculations. The charge-neutral layered perovskite of [Ce^IV^Ta_2_O_7_] was obtained through soft chemical oxidation of an anionic [Ce^III^Ta_2_O_7_]^−^ perovskite layer sheet with MnO_4_^−^ ions. TEM and AFM images were used to elucidate that the [Ce^IV^Ta_2_O_7_] particle had a nanoplate morphology with 10–30 stacked perovskite layers, similar to the anionic perovskite that was used as the precursor. The Ce and Ta L_3_-edge XANES spectra of [Ce^IV^Ta_2_O_7_] indicated that the cerium ion in the perovskite layer fully changed to a tetravalent ion and tantalum was maintained as a heptavalent ion due to the oxidation of [Ce^III^Ta_2_O_7_]^−^. The detailed analysis of the SAED pattern provides evidence that the Ox-form takes the higher symmetry of the tetragonal structure with no octahedral tilting, which is unique in conventional solid-state chemistry. It was presumed that this irregular stabilization of the tetrahedral symmetry in [CeTa_2_O_7_] is caused by the relaxation of the obvious displacement of Ta atoms to the outside of the perovskite layer. The detailed local structures around the Ce and Ta atoms were obtained based on the EXAFS spectra of each elemental absorption edge, and all bond lengths in the [Ce^III^Ta_2_O_7_]^−^ and [Ce^IV^Ta_2_O_7_] perovskites were found to be in good agreement with those obtained from the DFT calculations. Although the [Ce^IV^Ta_2_O_7_] perovskite maintained strong optical absorptions in the UV and blue-light regions, the strength of the NIR absorption at *ca*. 1 eV exhibited in the anionic [Ce^III^Ta_2_O_7_]^−^ perovskite was weakened. These changes in absorption properties should be ascribed to the fact that no MMCT transition of Ce 4f → Ta 5d occurred because, based on DOS calculations, the 4f electron associated with the oxidation of cerium disappeared. Furthermore, the obtained [Ce^IV^Ta_2_O_7_] perovskite layer can be reduced by reaction with ascorbic acid solution to form the anionic [Ce^III^Ta_2_O_7_]^−^ perovskite layer, which was confirmed by XRD and XANES analyses. In addition, cyclic voltammetry in acetonitrile revealed that the [Ce^III^Ta_2_O_7_] perovskite exhibits a chemically reversible redox behavior. The redox ability in the layered perovskite can form a new material group for catalyst science, and the Ce^4+^-based charge-neutral perovskite could be applied to polar materials based on their structural features. This work successfully defined the possibility of achieving extraordinary redox performance in layered perovskites, which could lead to the definition of an innovative material family that contributes to the further development of perovskite compounds through precise control of the electronic structure associated with optical performance. Particularly, the new concept of layer charge control and charge-neutral layered perovskite materials will be effectively applied to the materials science field, such as electrochromic materials utilizing color change by redox reactions and polar materials using BO_6_ octahedral distortion.

## Data availability

All experimental data, computational data and detail experimental procedure are provided in the ESI.†

## Author contributions

T. Hasegawa: all experiments except for the electrochemical analysis, writing-original draft, supervision, conceptualization. N. Yamasaki: measurement of the electrochemical analysis. T. Ueda supervision for the electrochemical analysis. Y. Asakura and S. Yin: writing-review and editing.

## Conflicts of interest

There are no conflicts to declare.

## Supplementary Material

SC-012-D1SC03053A-s001
