# Supplementary material for: Ce(iv)-centered charge-neutral perovskite layers topochemically derived from anionic [CeTa2O7]− layers
Source: Chem Sci. 2021 Oct 15;12(45):15016–27. doi: 10.1039/d1sc03053a (PMC8612395; doi:10.1039/d1sc03053a)
Supplement: SC-012-D1SC03053A-s001 [file SC-012-D1SC03053A-s001.pdf]

Electronic Supplementary Material (ESI) for Chemical Science.
This journal is © The Royal Society of Chemistry 2021

Electronic supplementary information (ESI) for:

Ce(IV)-centered charge-neutral perovskite layers topochemically-derived from anionic $[CeTa_2O_7]^-$ layer

Takuya Hasegawa,[1†], Naoki Yamasaki,[2] Yusuke Asakura[1], Tadaharu Ueda,[2,3] and Shu Yin[1]

[1] Institute of Multidisciplinary Research for Advanced Material (IMRAM), Tohoku University, 2-1-1 Katahira, Aoba-ku, Sendai, 980-8577, Japan
[2] Department of Marine Resources Science, Faculty of Agriculture and Marine Science, Kochi University, Nankoku, 783-8502, Japan
[3] Center for Advanced Marine Core Research, Kochi University, Nankoku 783, 8520, Japan

†Corresponding author:

Assistant Professor Dr. Takuya Hasegawa

Tel/Fax +81-22-217-5599, E-mail: hase@tohoku.ac.jp

Content

1. Detailed experimental procedures
2. Figure S1 Linear combination fitting analysis of the Ce L_3-edge XANES spectra for all forms.
3. Figure S2 Comparison of the Ta L_3-edge XANES spectra of the $RbLaTa_2O_7$, Ta_2O_5 and TaS_2 references.
4. Figure S3 SEM images for all forms.
5. Figure S4 Low-magnification HAADF-STEM images in the basal and vertical directions for the Ox-form.
6. Figure S5 ABF-STEM in the basal and vertical directions for the Ox-form.
7. Figure S6 STEM-EDS mappings of the Ox-form: HAADF-STEM, O *K*-, Ce *L*-, Ta *L*-edges.
8. Figure S7 XRD patterns of the Rb-, H- and EA-forms.
9. Figure S8 ^{1}H MAS NMR spectra for the H-form and $HLaTa_2O_7$.
10. Figure S9 TG curves with a heating rate is 10 °C/min. of the (A) H-, (B) TBA-and Ox-forms.
11. Figure S10 Measured SAED patterns for the TBA- and Ox-forms and the simulated SAED pattern for $RbCeTa_2O_7$.
12. Figure S11 (A) Simulated crystal structure with electron-density mapping via yellow isosurfaces at 0.18 $Å^{-3}$ for the (left) anionic $[Ce^{III}Ta_2O_7]^-$ and (right) charge-neutral $[Ce^{IV}Ta_2O_7]$ perovskite sheets.
13. Figure S12 (A) Ce L_3- and (B) Ta L_3-edge k^3-weighted EXAFS spectra for the TBA- and Ox-forms.
14. Figure S13 (A) Ce L_3- and (B) Ta L_3-edge EXAFS spectra in *q*-space for the TBA- and Ox-forms.
15. Figure S14 (A) Ce L_3- and (B) Ta L_3-edge EXAFS spectra in *R*-space for all forms.
16. Figure S15 Electronic band structure of (A) anionic $[Ce^{III}Ta_2O_7]^-$ and (B) charge-neutral $[Ce^{IV}Ta_2O_7]$ perovskite layers.
17. Figure S16 (A) Cyclic voltammograms of the PDDA/$Ce^{III}Ta_2O_7$/PEI-Au electrode at first to third cycles, and (B) bare Au and PEI coated-Au electrodes in in acetonitrile containing 100 mM $TBAPF_6$ at scan rate of 100 mV/s.
18. Table S1 Crystallographic parameters of the anionic $[Ce^{III}Ta_2O_7]^-$ and charge-neutral $[Ce^{IV}Ta_2O_7]$ perovskite layers optimized by DFT calculation.
19. Table S2 Refined parameters of EXAFS fitting for TBA- and Ox-forms.
20. Table S3 CIE chromatic coordination values of the TBA- and Ox-forms.
21. References

Detailed experimental procedures

Synthesis of the $RbCeTa_2O_7$ host material. The $RbCeTa_2O_7$ host (Rb-form) was synthesized by the polymerized complex (PC) method according to the literature.[1] Anhydrous citric acid (270 mmol) was dissolved in 300 mL MeOH during stirring, followed by the addition of $TaCl_5$ powder (30 mmol). Then, $Ce(NO_3)_3 \cdot 6H_2O$ (15 mmol) and $RbNO_3$ (22.5 mmol) were dissolved in a Ta-containing citrate MeOH solution, which was stirred at 80°C for 2 h. Subsequently, EG (1.08 mol) was added to the solution while the temperature was maintained. After stirring for a few minutes, the solution was heated at 200°C overnight to obtain the polymerized gel. The organic components in the transparent gel were decomposed by heating at 400°C using a mantle heater until a yellowish-colored powder formed. After cooling, the calcined powder was preheated at 600°C for 24 h in the ambient atmosphere and then reground using an agate mortar to form the homogeneous precursor. After the fine precursor was mixed with graphite powder with a molar ratio of Ce:graphite=1:0.5, the mixture powder was finally heated at 1100°C for 5 h in N_2 gas with a flow rate of 100 mL/min. The heating temperature was changed from the literature (1000°C) in order to obtain the higher crystallinity particles. The obtained pale green powder was washed with deionized water and EtOH, filtered and dried at 60°C in vacuo until sufficient drying was achieved.

Material Characterization. The crystal phases of all of the obtained $[CeTa_2O_7]$ derivatives were determined through X-ray diffraction (XRD) using an X-ray diffractometer (D2 PHASER, Bruker) with CuKα radiation (λ=1.54059 Å). The morphologies of the bulk samples were observed through field-emission scanning electron microscopy (FE-SEM; JSM-7800F, JEOL), and transmission electron microscopy (TEM) images and selected area electron diffraction (SAED) images were taken by a JEM-2100F, JEOL. The thicknesses and morphologies of the delaminated sheets were observed by atomic force microscopy (AFM) using an SII Nanocute (SII Technology). For TEM observation of the cleaned Si wafer that was used for AFM observation, the delaminated sample was mounted onto the Cu grid by drop casting a dilute nanosheet dispersion EtOH solution and was left to stand under ambient conditions for a few hours. An atomic resolution analytical electron microscope (JEM-ARM200F, JEOL) equipped with a spherical aberration corrector with a 200 kV acceleration voltage was used for the observation of the high-angle annular dark field (HAADF) and annular bright field (ABF) scanning transmission electron microscope (STEM) images. Solid-state ^1H magic-angle spinning nuclear magnetic resonance (MAS NMR) spectra were

measured with an ECA-300 spectrometer (JEOL RESONANCE Inc.) through the depth-pulse sequence at room temperature and a spinning rate of 10 kHz; the corresponding data were obtained with 400 times integration. To understand the chemical state and local structure, Ce and Ta L_3-edge X-ray absorption fine structure (XAFS) of the obtained [$CeTa_2O_7$] derivative samples and reference samples, which were prepared with boron nitride powder and pelletized, were measured. This measurement was conducted using a transmission mode by the beamline BL12C in the Photon Factory (PF) at High Energy Accelerator Research Organization, Japan (KEK). The obtained X-ray absorption near edge structure (XANES) and extended X-ray absorption fine structure (EXAFS) spectra were analyzed by Athena and Artemis programs.[2] The surface chemical state of the samples was measured with X-ray photoelectron spectroscopy (XPS; PHI5600, ULVAC-PHI Inc.) using monochromatized X-rays of AlKα (300 W). The binding energy of the XPS measurement was calibrated with the C 1s level at 284.6 eV. The optical absorption properties of the samples were measured using an ultraviolet-visible-infrared (UV-vis-IR) spectrometer (V-700, JASCO Co.) with an integrated sphere attachment to obtain diffuse reflectance (DR) spectra. The thermal stability of the [$CeTa_2O_7$] derivative samples was characterized under ambient air with a flow rate of 200 mL/min by a thermogravimetric-differential thermal analyzer (TG-DTA; ThemoPlus TG 8120, Rigaku).

Theoretical calculation. Theoretical calculations were performed with the Quantum Espresso program.[3] The layered perovskite structure, with a 12 Å vacuum layer inserted in the interlayer, was optimized for the atomic positions that retained the tetragonal crystal system. A generalized gradient approximation (GGA) exchange-correlation functional pseudopotential, which was parametrized by the Perdew-Burke-Ernzerhof revised form for solids (PBEsol) for the structural optimizations and the PBEsol+U with U(Ce,Ta)= (1 eV, 3 eV) for the electronic structure,[4] was used for all calculations. In these calculations, the anionic [$Ce^{III}Ta_2O_7$]$^-$ and charge-neutral [$Ce^{IV}Ta_2O_7$] layers were performed with several parameters including a plane-wave cutoff energy of 50 Ry and a 6×6×1 grid Monkhorst–Pack k-point mesh. The following factors were considered in the theoretical calculations: the effect of strong electron correlations in the Ce 4f and Ta 5d orbitals, an energy convergence of 1.0×10^{-6} eV/atom and a maximum force tolerance of 0.01 eV/Å. The crystal structure of $RbCeTa_2O_7$ was utilized to optimize the atomic positions and lattice parameters with the BFGS algorithm. The total charge was set to -1 for the anionic [$Ce^{III}Ta_2O_7$]$^-$ layer and zero for the charge-neutral [$Ce^{IV}Ta_2O_7$] layer in all calculations.

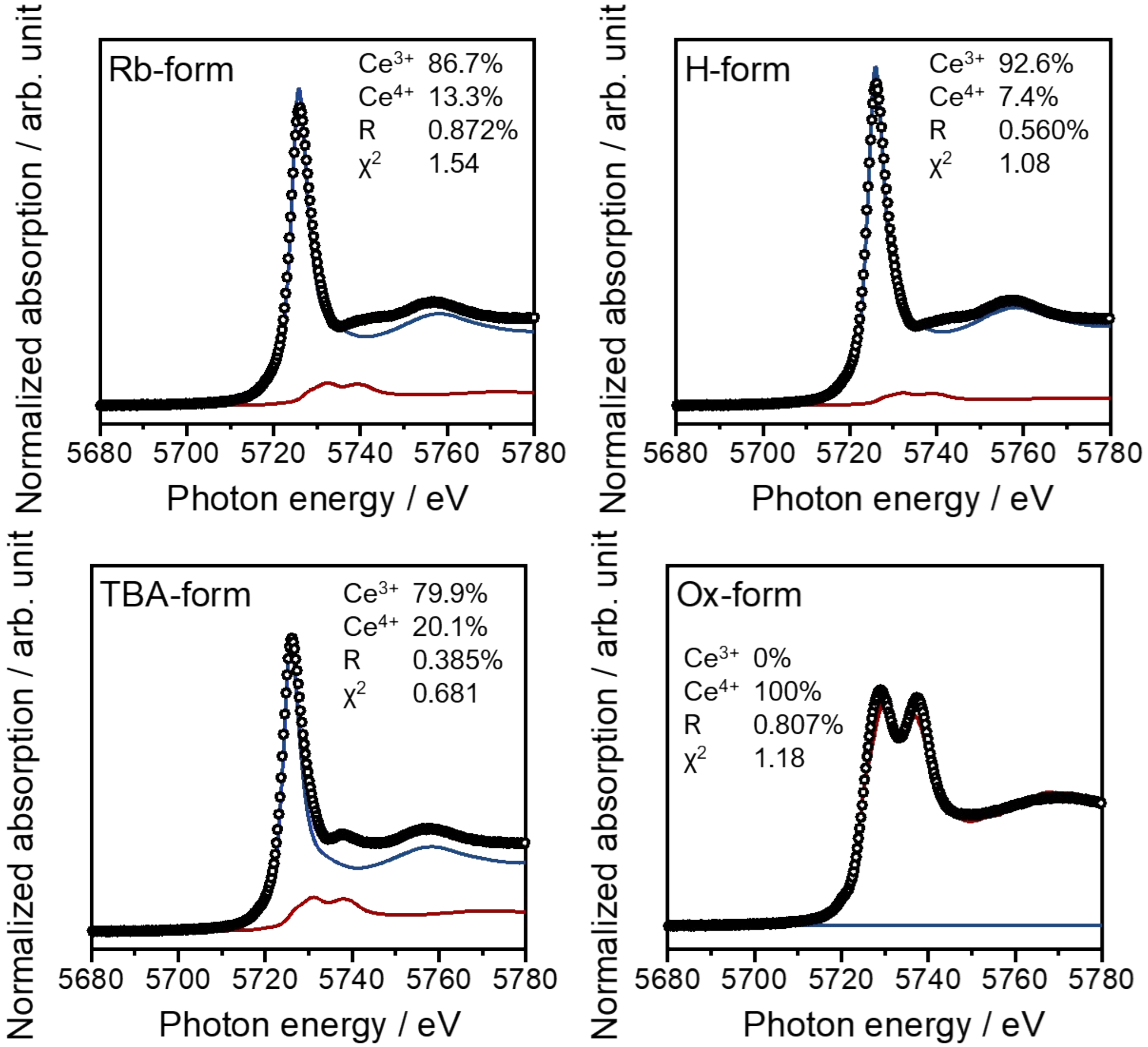

Figure S1 Linear combination fitting analysis of the Ce L_3-edge XANES spectra for all forms.

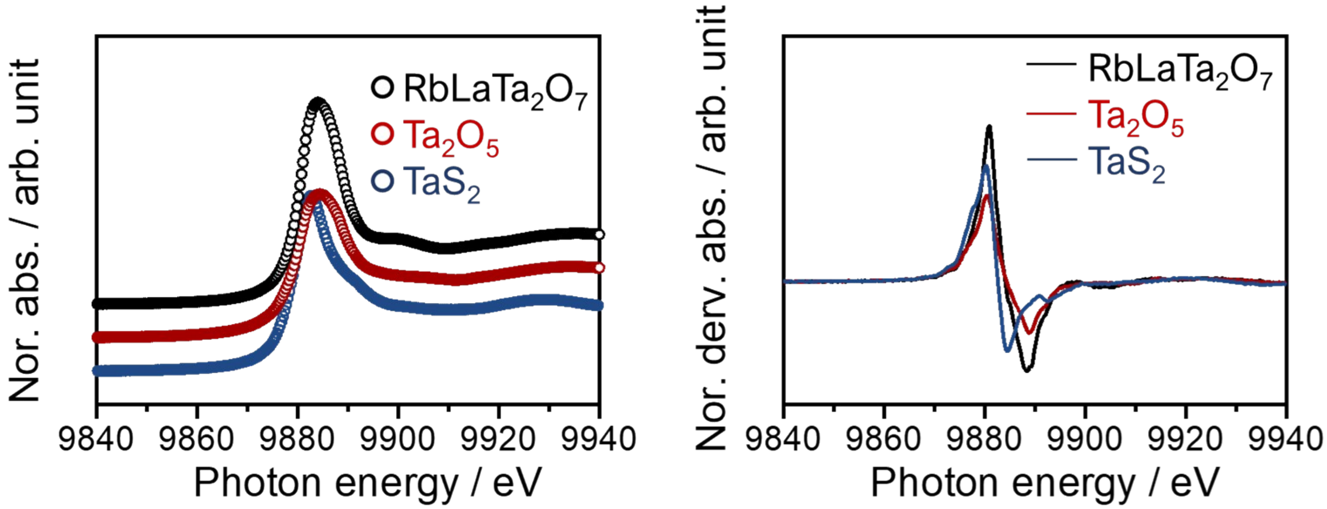

Figure S2 Comparison of the Ta L_3-edge XANES spectra of the $RbLaTa_2O_7$, Ta_2O_5 and TaS_2 references.

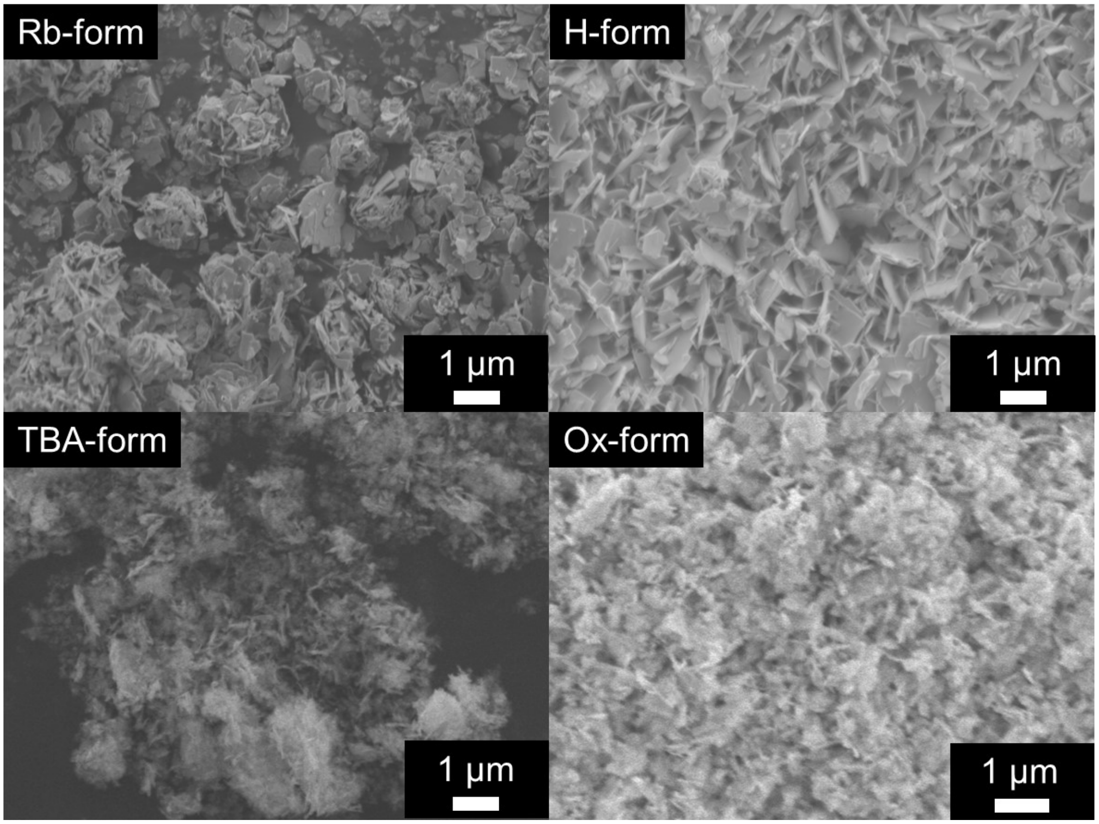

Figure S3 SEM images for all forms.

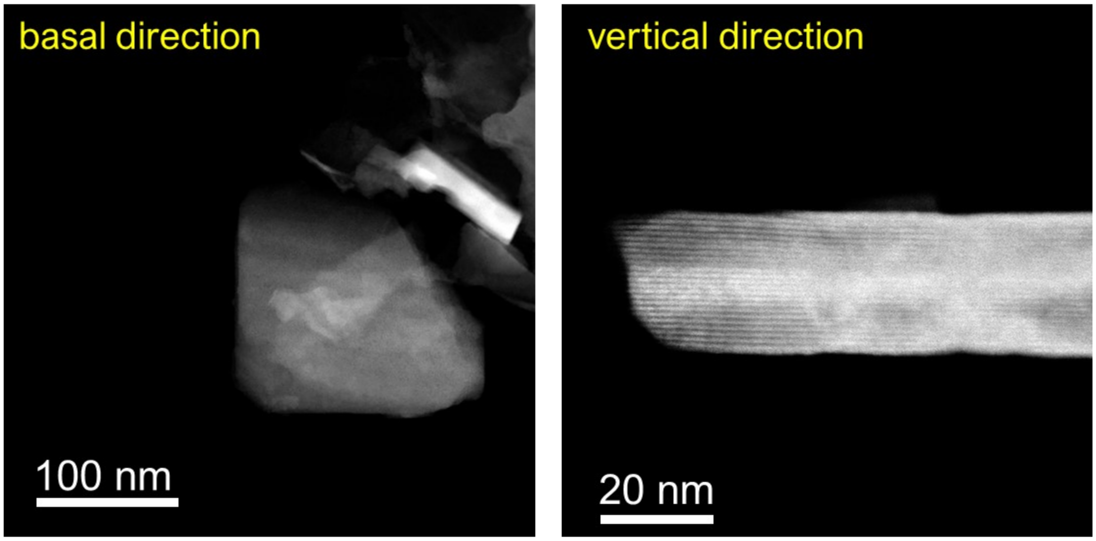

Figure S4 Low-magnification HAADF-STEM images in the basal and vertical directions for the Ox-form.

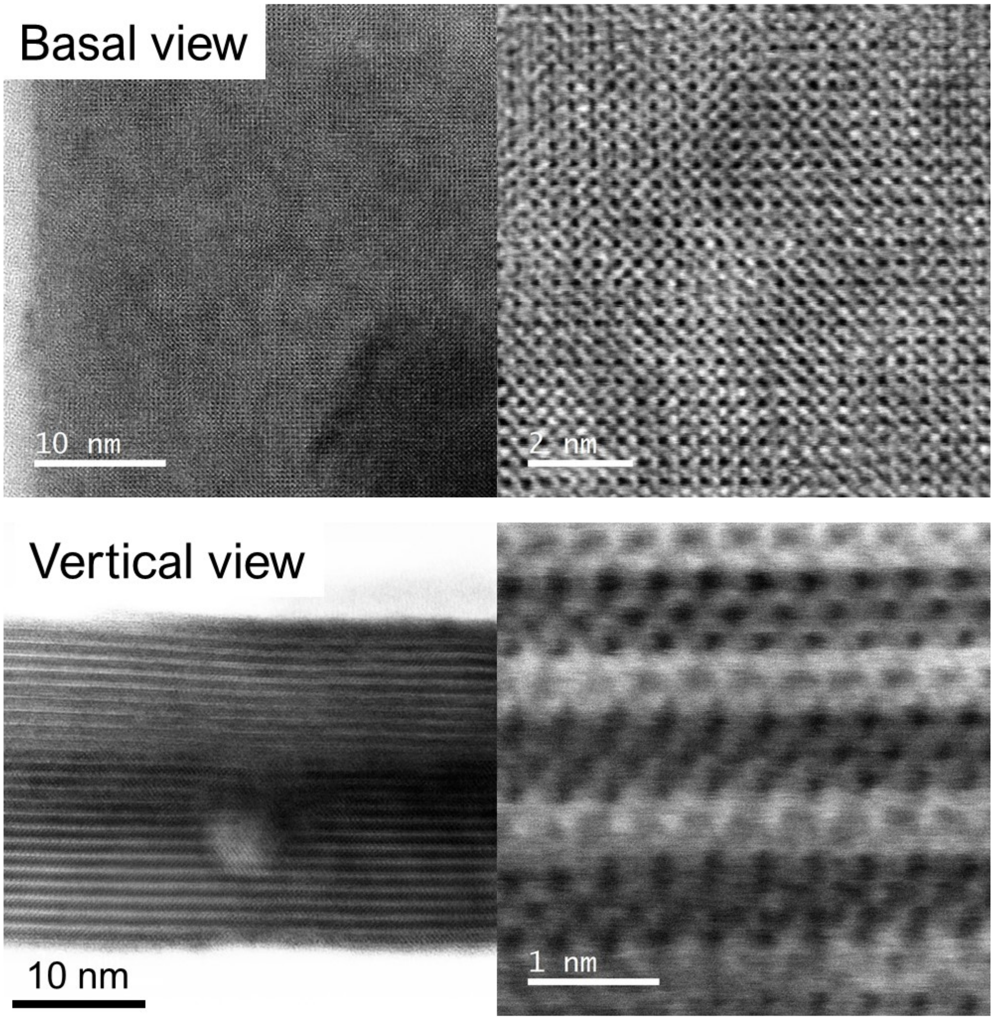

Figure S5 ABF-STEM in the basal and vertical directions for the Ox-form.

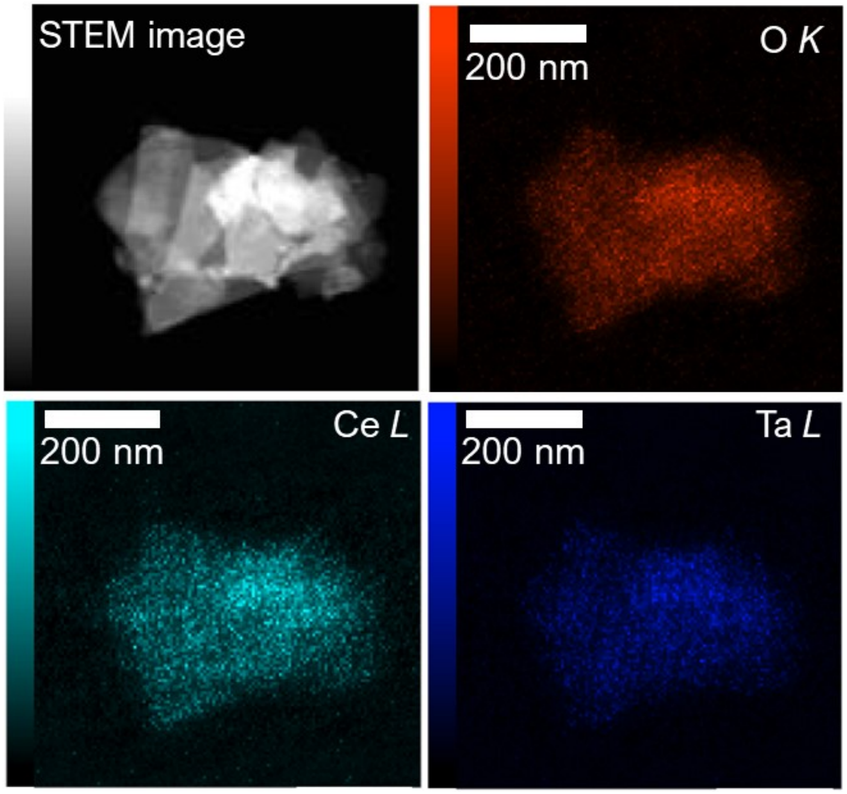

Figure S6 STEM-EDS mappings of the Ox-form: HAADF-STEM, O *K*-, Ce *L*-, Ta *L*-edges.

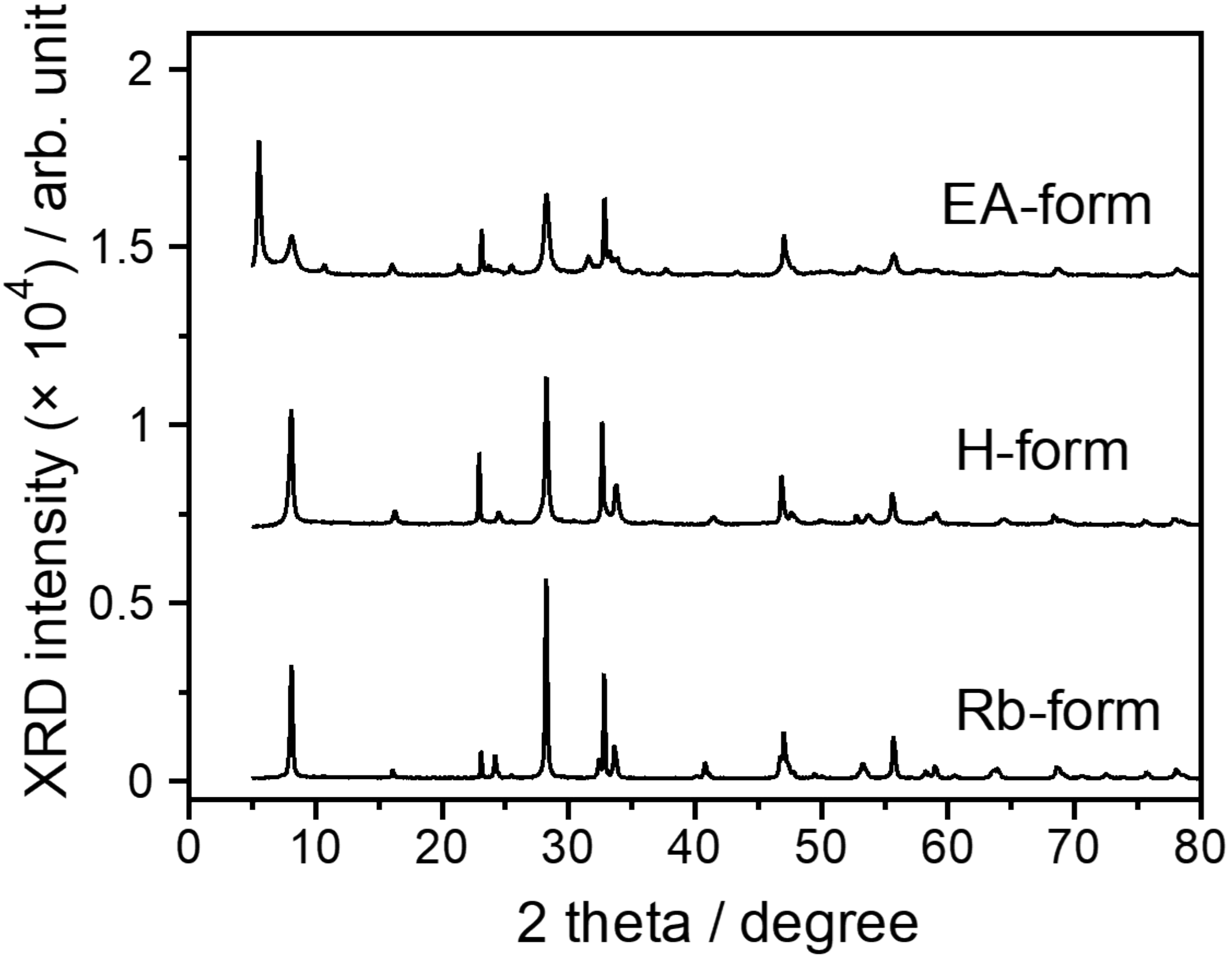

Figure S7 XRD patterns of the Rb-, H- and EA-forms.

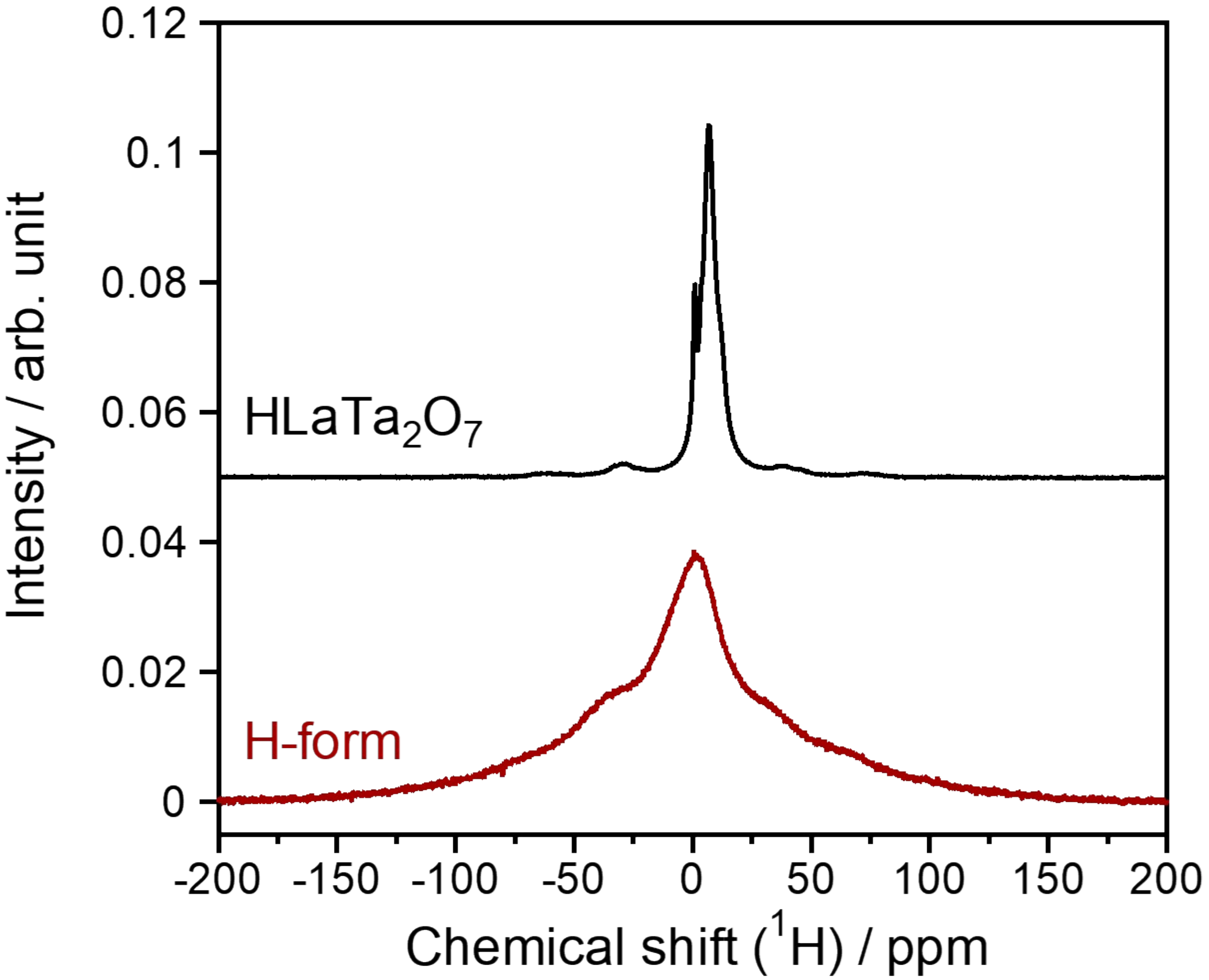

Figure S8 ^{1}H MAS NMR spectra for the H-form and $HLaTa_2O_7$.

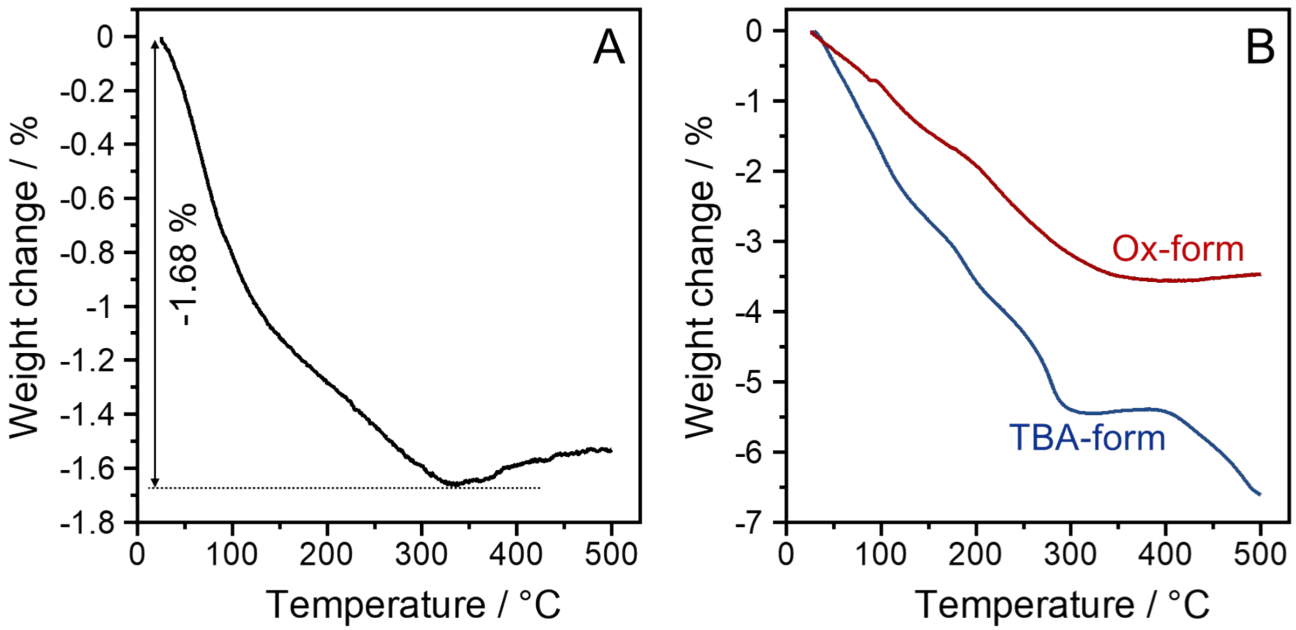

Figure S9 TG curves with a heating rate is 10 °C/min. of the (A) H-, (B) TBA-and Ox-forms.

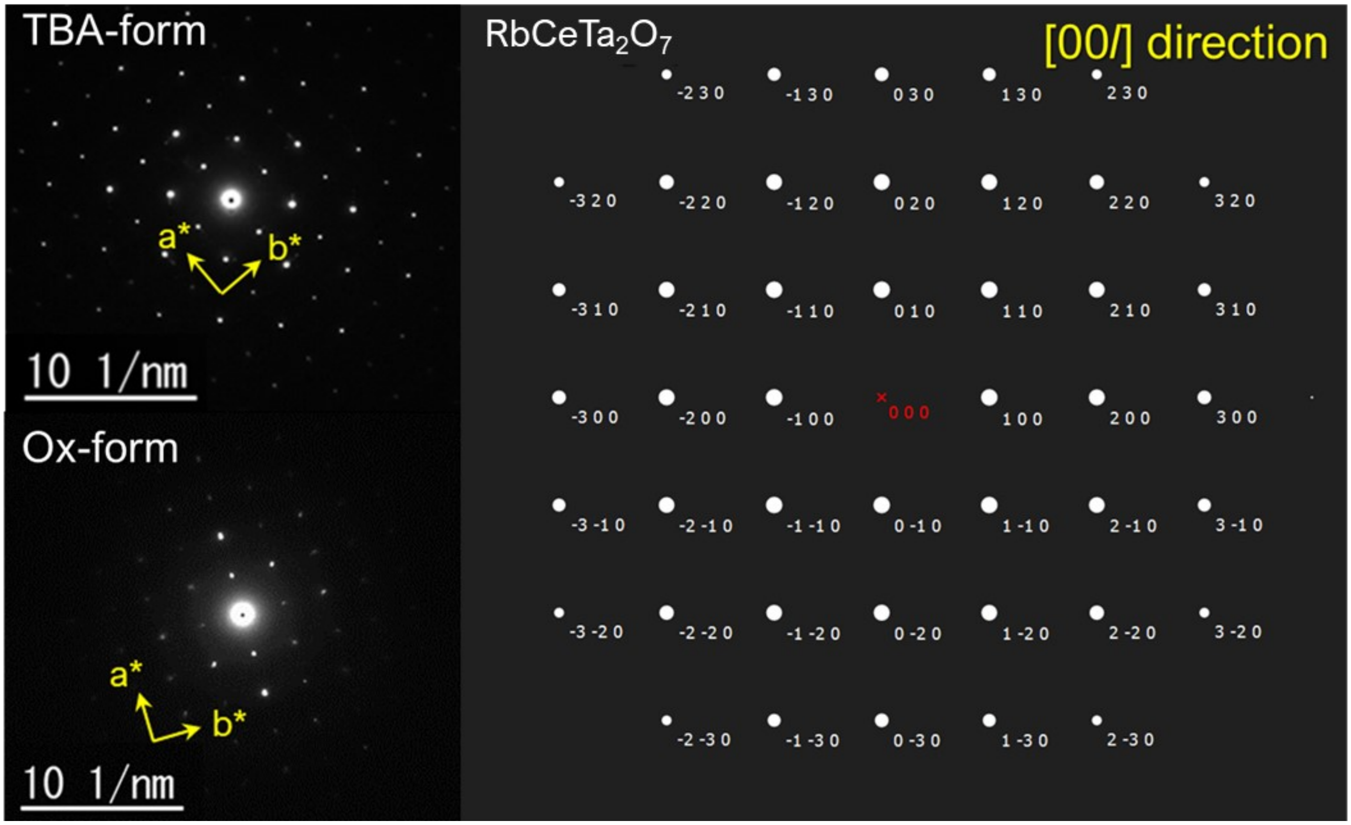

Figure S10 Measured SAED patterns for the TBA- and Ox-forms and the simulated SAED pattern for RbCeTa$_2$O$_7$.

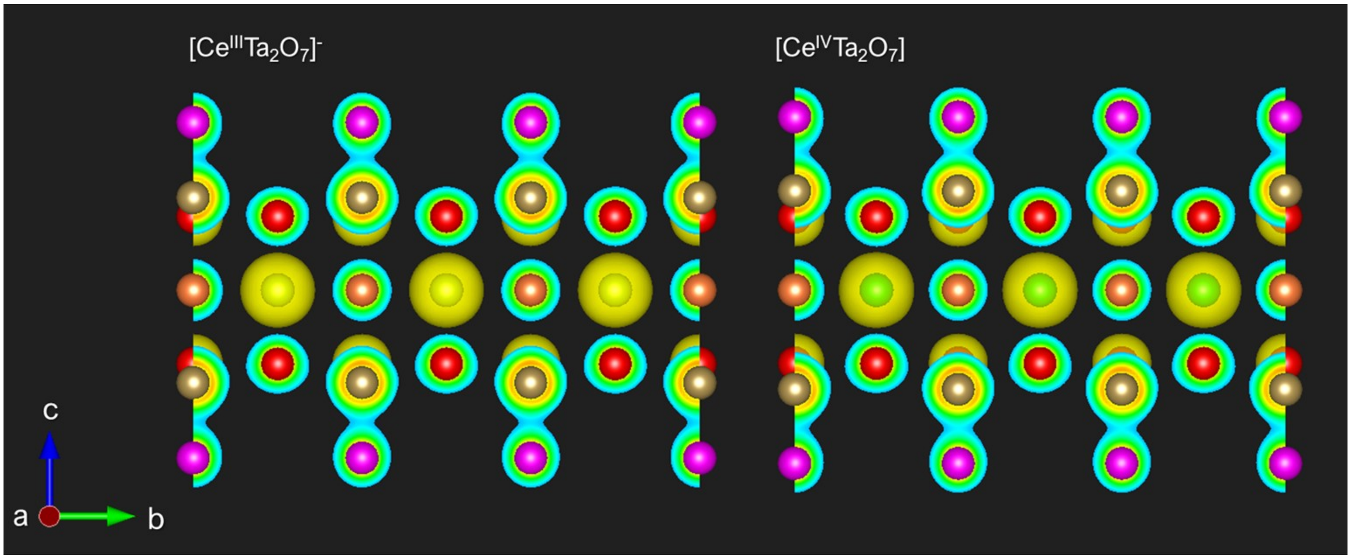

Figure S11 (A) Simulated crystal structure with electron-density mapping via yellow isosurfaces at 0.18 Å^{-3} for the (left) anionic [Ce^{III}Ta$_2$O$_7$]$^-$ and (right) charge-neutral [Ce^{IV}Ta$_2$O$_7$] perovskite sheets.

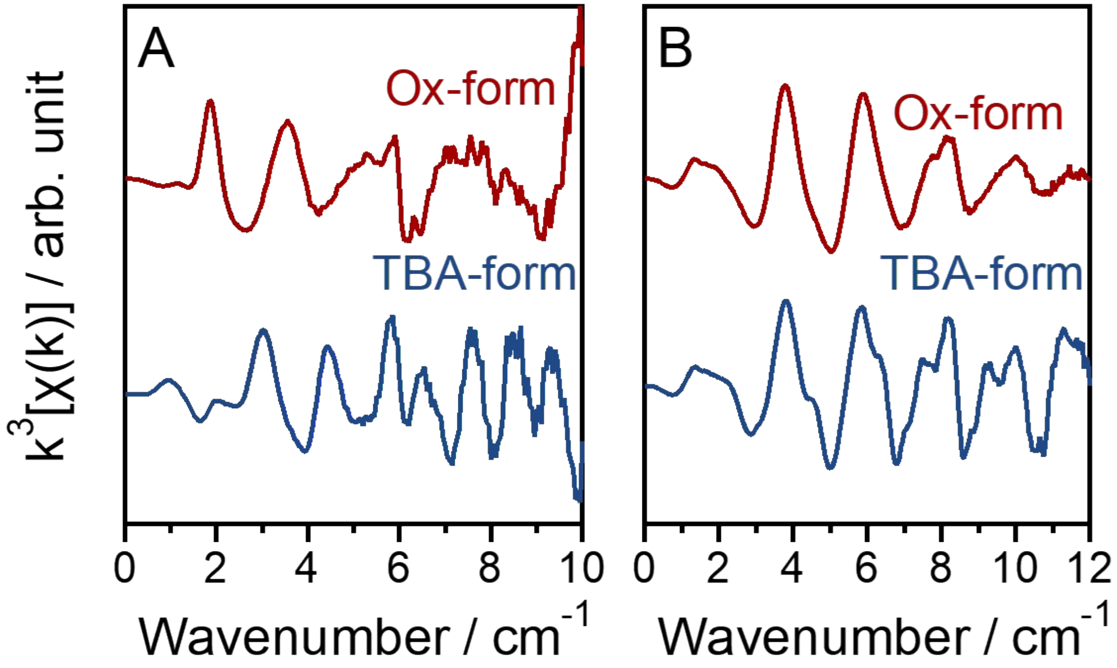

Figure 12 (A) Ce L_3- and (B) Ta L_3-edge k^3-weighted EXAFS spectra for the TBA- and Ox-forms.

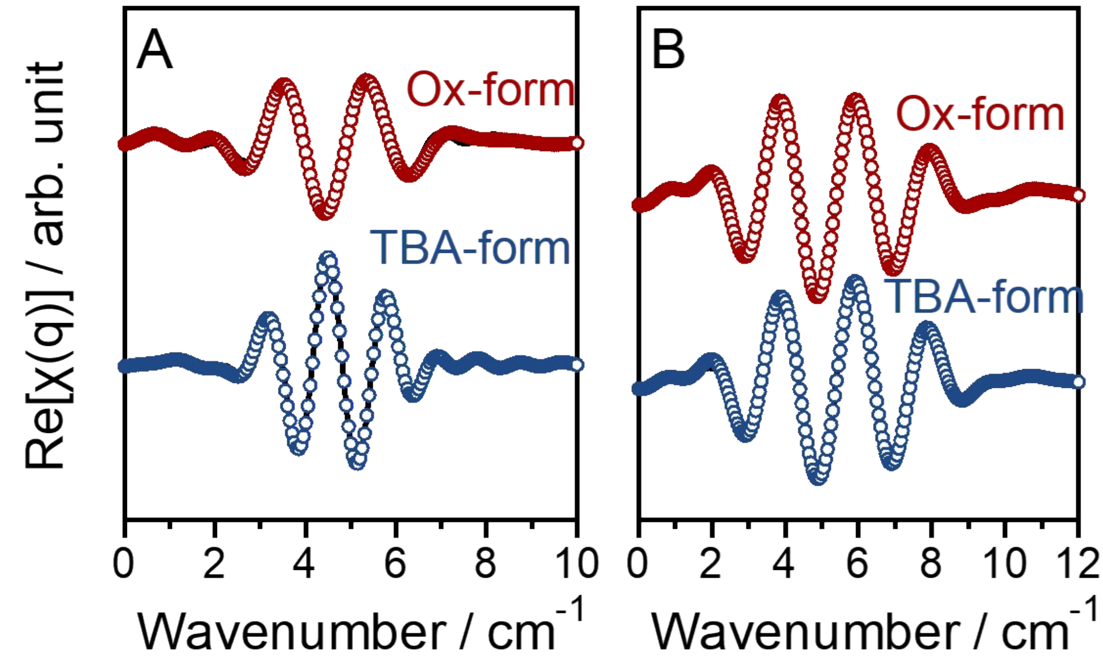

Figure 13 (A) Ce L_3- and (B) Ta L_3-edge EXAFS spectra in q-space for the TBA- and Ox-forms.

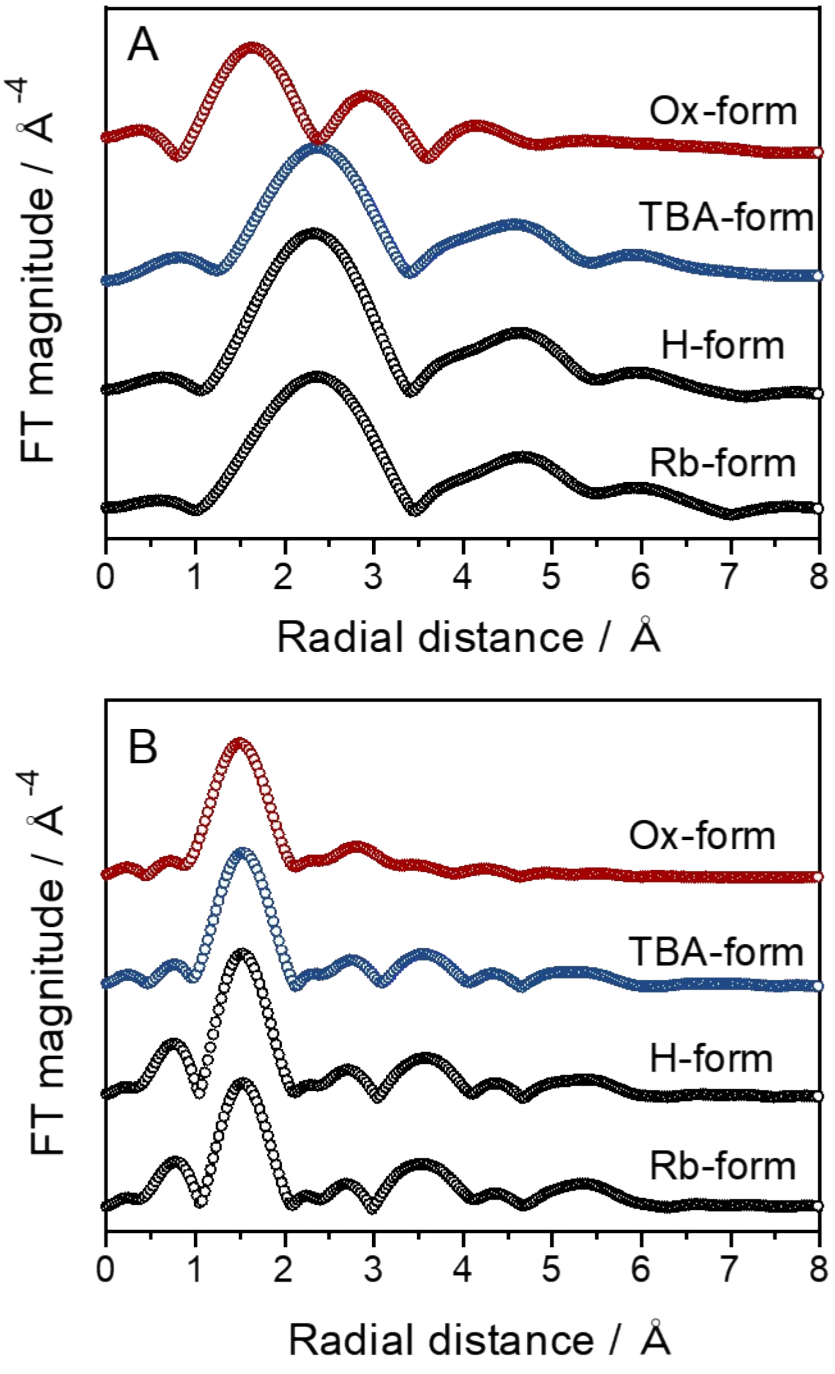

Figure S14 (A) Ce L_3- and (B) Ta L_3-edge EXAFS spectra in R-space for all forms.

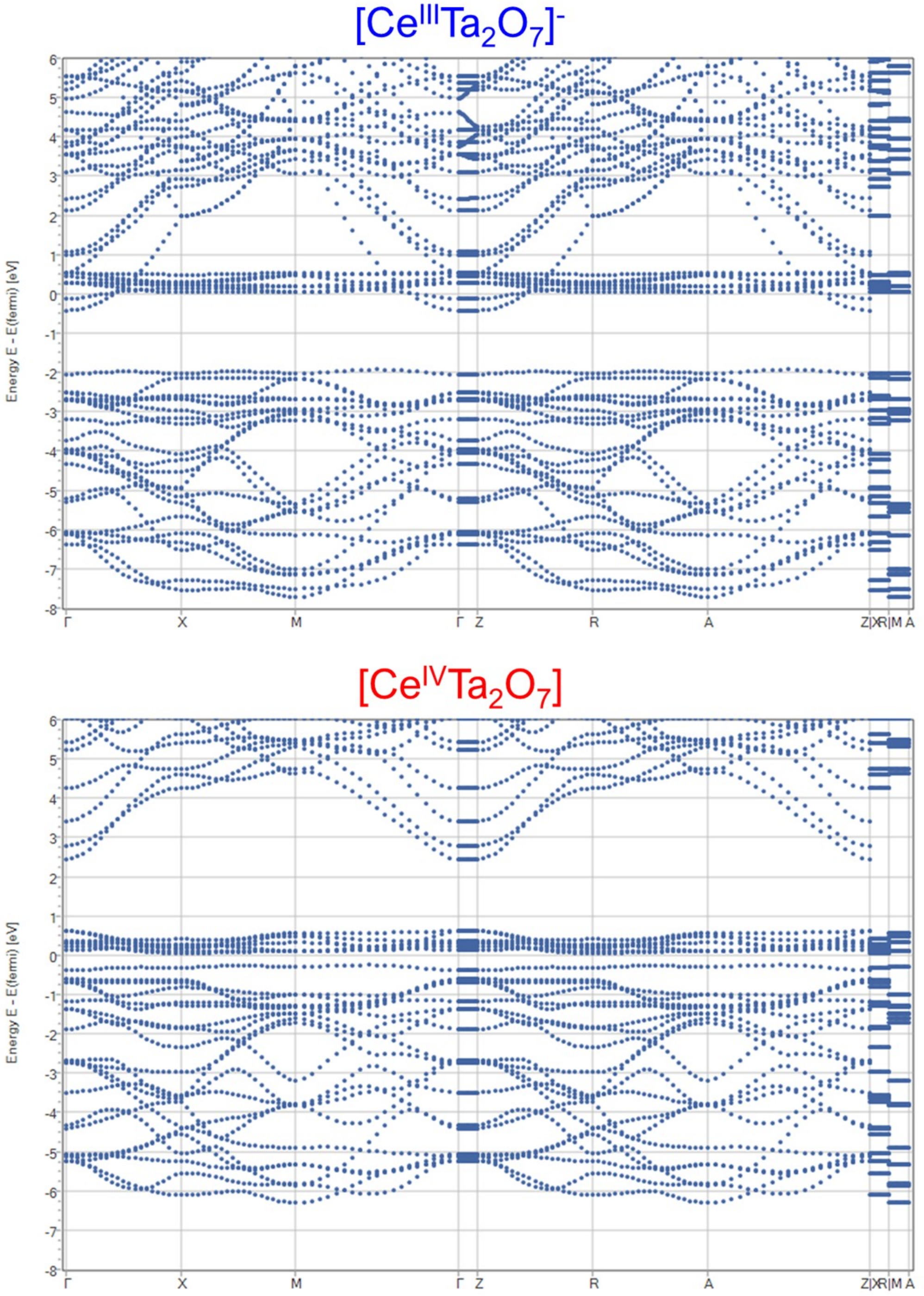

Figure S15 Electronic band structure of (A) anionic $[Ce^{III}Ta_2O_7]^-$ and (B) charge-neutral $[Ce^{IV}Ta_2O_7]$ perovskite layers.

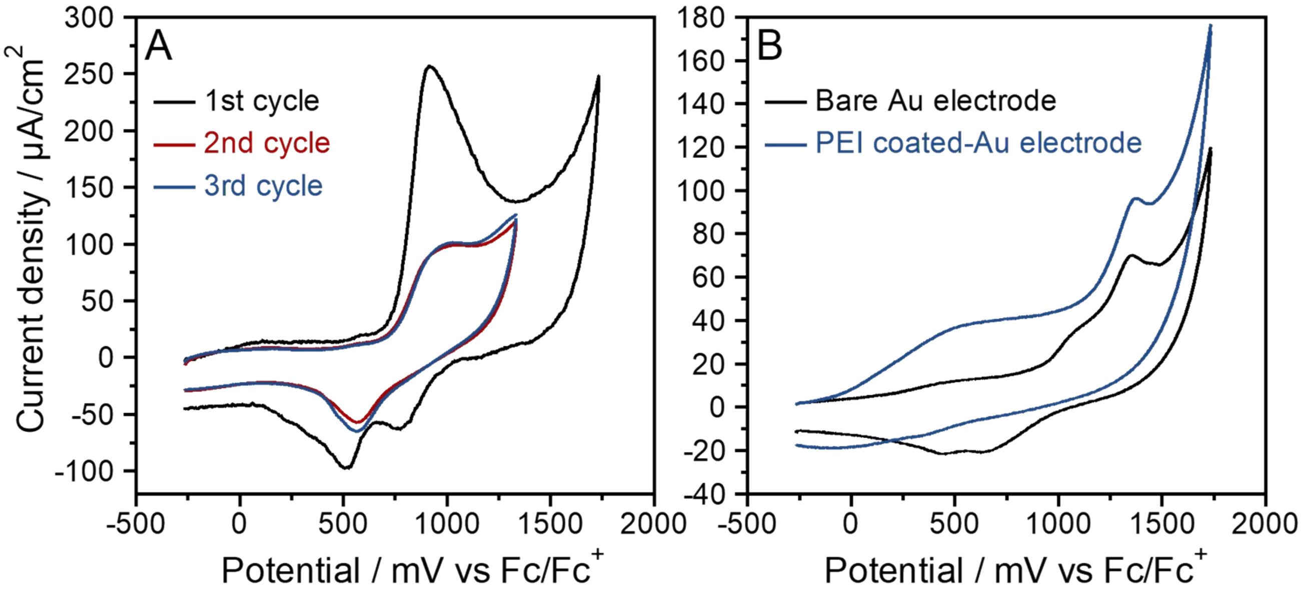

Figure S16 (A) Cyclic voltammograms of the PDDA/$Ce^{III}Ta_2O_7$/PEI-Au electrode at first to third cycles, and (B) bare Au and PEI coated-Au electrodes in acetonitrile containing 100 mM $TBAPF_6$ at scan rate of 100 mV/s.

Table S1 Crystallographic parameters of the anionic $[Ce^{III}Ta_2O_7]^-$ and charge-neutral $[Ce^{IV}Ta_2O_7]$ perovskite layers optimized by DFT calculation.

Perovskite layer			$[Ce^{III}Ta_2O_7]^-$		$[Ce^{IV}Ta_2O_7]$	
Crystal system			Tetragonal			
Space group			$P4/mm$ (99)			
a=b (Å)			3.94932		3.82032	
c (Å)			23.09821		22.30364	
Atomic parameters						
Atom (Wyckoff)	x	y	z	BVS	z	BVS
Ce1 (1b)	1/2	1/2	0.250000	2.9417	0.250000	3.5203
Ta1 (1a)	0	0	0.343721	5.0750	0.353849	5.2086
Ta2 (1a)	0	0	0.156279	5.0750	0.146156	5.2096
O1 (2c)	1/2	0	0.324733	-	0.326716	-
O2 (2c)	1/2	0	0.175267	-	0.173286	-
O3 (1a)	0	0	0.420041	-	0.431364	-
O4 (1a)	0	0	0.079959	-	0.068636	-
O5 (1a)	0	0	0.250000	-	0.250003	-

Table S2 Refined parameters of EXAFS fitting for TBA- and Ox-forms.

Derivatives	Path	N	Amp	E_0	R (Å)	σ^2	R-factor
TBA-form	Ce-O2	8	0.923	11.420	2.646(4)	0.0071(7)	0.149%
	Ce-O3	4	1.08	-2.755	2.749(6)	0.0001(9)	
	Ta-O1	1	2.101	-21.505	1.78(3)	0.012(6)	1.60%
	Ta-O2	4	0.946	9.888	2.002(6)	0.009(1)	
	Ta-O3	1	1.069	-12.553	2.26(3)	0.007(5)	
Ox-form	Ce-O2	8	0.918	-28.294	2.53(1)	0.012(3)	1.77%
	Ce-O3	4	0.954	-28.534	2.70(5)	0.017(8)	
	Ta-O1	1	0.984	-7.926	1.75(8)	0.01(2)	0.546%
	Ta-O2	4	1.005	4.608	1.95(1)	0.008(2)	
	Ta-O3	1	1.408	-26.906	2.36(10)	0.01(2)	

Table S3 CIE chromatic coordination values of the TBA- and Ox-forms.

	TBA-form	Ox-form
L^*	65.52	67.38
a^*	-4.69	-4.04
b^*	11.84	18.65

References

(1) Hasegawa, T.; Shigee, A.; Nishiwaki, Y.; Nagasako, M.; Hanindriyo, A. T.; Hongo, K.; Maezono, R.; Ueda, T.; Yin, S. New Layered Perovskite Family Built from $[CeTa_2O_7]^-$ Layers: Coloring Mechanism from Unique Multi-Transitions. *Chem. Commun.* **2020**, *56*, 8591–8594.

(2) Ravel, B.; Newville, M. ATHENA, ARTEMIS, HEPHAESTUS: Data Analysis for X-Ray Absorption Spectroscopy Using IFEFFIT. *J. Synchrotron Radiat.* **2005**, *12*, 537–541.

(3) Giannozzi, P.; Baroni, S.; Bonini, N.; Calandra, M.; Car, R.; Cavazzoni, C.; Ceresoli, D.; Chiarotti, G. L.; Cococcioni, M.; Dabo, I.; et al. QUANTUM ESPRESSO: A Modular and Open-Source Software Project for Quantum Simulations of Materials. *J. Phys. Condens. Matter* **2009**, *21*.

(4) Perdew, J. P.; Ruzsinszky, A.; Csonka, G. I.; Vydrov, O. A.; Scuseria, G. E.; Constantin, L. A.; Zhou, X.; Burke, K. Restoring the Density-Gradient Expansion for Exchange in Solids and Surfaces. *Phys. Rev. Lett.* **2008**, *100*, 136406.
